# Blessing and curse of bioclimatic variables: A comparison of different calculation schemes and datasets for species distribution modeling within the extended Mediterranean area

**DOI:** 10.1002/ece3.10553

**Published:** 2023-09-28

**Authors:** Christian Merkenschlager, Freddy Bangelesa, Heiko Paeth, Elke Hertig

**Affiliations:** ^1^ Regional Climate Change and Health, Faculty of Medicine University of Augsburg Augsburg Germany; ^2^ Institute of Geography and Geology Universtiy of Wuerzburg Wuerzburg Germany

**Keywords:** BIOCLIM, bioclimatic variables, CRU, ecological niche modeling, E‐OBS, ERA5‐Land, Mediterranean, WorldClim

## Abstract

Bioclimatic variables (BCVs) are the most widely used predictors within the field of species distribution modeling, but recent studies imply that BCVs alone are not sufficient to describe these limits. Unfortunately, the most popular database, WorldClim, offers only a limited selection of bioclimatological predictors; thus, other climatological datasets should be considered, and, for data consistency, the BCVs should also be derived from the respective datasets. Here, we investigate how well the BCVs are represented by different datasets for the extended Mediterranean area within the period 1970–2020, how different calculation schemes affect the representation of BCVs, and how deviations among the datasets differ regionally. We consider different calculation schemes for quarters/months, the annual mean temperature (BCV‐1), and the maximum temperature of the warmest month (BCV‐5). Additionally, we analyzed the effect of different temporal resolutions for BCV‐1 and BCV‐5. Differences resulting from different calculation schemes are presented for ERA5‐Land. Selected BCVs are analyzed to show differences between WorldClim, ERA5‐Land, E‐OBS, and CRU. Our results show that (a) differences between the two calculation schemes for BCV‐1 diminish as the temporal resolution decreases, while the differences for BCV‐5 increase; (b) with respect to the definition of the respective month/quarter, intra‐annual shifts induced by the calculation schemes can have substantially different effects on the BCVs; (c) all datasets represent the different BCVs similarly, but with partly large differences in some subregions; and (d) the largest differences occur when specific month/quarters are defined by precipitation. In summary, (a) since the definition of BCVs matches different calculation schemes, transparent communication of the BCVs calculation schemes is required; (b) the calculation, integration, or elimination of BCVs has to be examined carefully for each dataset, region, period, or species; and (c) the evaluated datasets provide, except in some areas, a consistent representation of BCVs within the extended Mediterranean region.

## Graphical abstract

Bioclimatic variables (BCVs) are one of the most widely used predictor variables in ecological niche modeling, but they are not subject to a uniform calculation base. Furthermore, recent literature recommends the use of more (bio)climatological variables than those provided by the most popular database, WorldClim. Therefore, we analyzed different calculation schemes of BCVs and gridded station‐based observational or reanalysis weather (GSORW) datasets with respect to regional deviations within the extended Mediterranean region for the period 2000–2020.

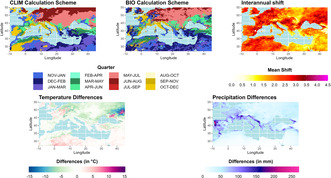

## 1 INTRODUCTION

Bioclimatic variables (BCVs) are the most commonly used predictors in species distribution modeling (SDM; Fourcade et al., 2018) to describe niche evolution (Warren et al., 2010), the delineation of protected areas (Esselman & Allan, 2011), the ecological limits (Cunze et al., 2016), and the habitat suitability of invasive species (Ibáñez‐Justicia et al., 2020; Koch et al., 2016). The BCVs contain information on temperature and precipitation at different temporal scales (e.g., annual, quarterly, monthly means, or sums), which are in these fields of research more relevant than monthly time series of climate data (Deblauwe et al., 2016) because they are physiologically meaningful for species distributions (Title & Bemmels, 2018). O'Donnell and Ignizio (2012) note that BCVs better represent the types of seasonal trends inherent in the physiological limitations of different species.

The initial set of 12 BCVs came with the start of the BIOCLIM program in 1984, which represents the beginning of modern SDM (Booth, 2018). The original set of BCVs was derived from monthly mean values of daily minimum and maximum temperatures and mean monthly precipitation sums. Although the first documentation of these variables was provided by Booth (1985) and Prendergast and Hattersley (1985), the BCVs can be traced back to Nix (1986). In 1996, BCVs were expanded to 19 variables by adding meaningful annual variables, such as the minimum temperature of the coldest month or the mean precipitation sums of the driest quarter (Booth, 2018). In 1999, additional variables were provided, including complex interactions associated with water balance calculations, bringing the total number of BCVs to 35 (Booth et al., 2014; Xu & Hutchinson, 2011).

The success story of BCVs within the SDM community dramatically increased with the release of freely downloadable global high‐resolution datasets (Fourcade et al., 2018). One of the most popular BCV datasets is provided by the WorldClim database (Fick & Hijmans, 2017; Hijmans et al., 2005), but other databases also provide BCVs (Bede‐Fazekas & Somodi, 2020; Booth, 2018). In addition, algorithms for calculating BCVs are available for several computing environments, such as the “biovars” function of the R package “dismo” (Biovars, Hijmans, 2006; Hijmans et al., 2022). The 19 BCVs provided by WorldClim are based on climate interpolation methods developed for the BIOCLIM program (Booth et al., 2014) and cover the entire land areas of the world except for Antarctica (Booth, 2022). The dataset is available in four different spatial resolutions (30 arc seconds–10 arc minutes, roughly 1–20 km) and provides means for the recent period 1970–2000 as well as historical and future scenarios (Title & Bemmels, 2018). Overall, WorldClim's 19 BCVs are the most used set of variables in SDM (Bradie & Leung, 2017). Booth et al. (2014) reviewed recent literature on maximum entropy models (MaxEnt) and found that more than 76% used at least one and 55% used all BCVs. This is also confirmed by the study of Fourcade et al. (2018), who reviewed 190 studies that modeled terrestrial organisms. Over 87% of these studies used at least one BCV, 20% used all BCVs, and over 42% used BCVs in addition to other variables.

The close relationship between climate and species distributions, the advances in modeling techniques, such as machine learning algorithms like MaxEnt and boosted regression trees (BRTs), and the frequent use of BCVs seem to be a blessing, since results from different studies on the same species seem to be comparable. However, the use of BCVs comes with caveats on several levels. First, many studies indicate that some of the BCVs are highly correlated (e.g., precipitation of the driest month [BCV‐14] and precipitation of the driest quarter [BCV‐17]), and most SDM techniques cannot deal with collinearity (e.g., Dormann et al., 2013; Fourcade et al., 2018; O'Donnell & Ignizio, 2012). Therefore, a preselection of relevant predictors should be made based on the species‐specific key limiting factors identified by expert knowledge or statistical approaches (Porfirio et al., 2014; Synes & Osborne, 2011; Title & Bemmels, 2018). This leads to the second problem, since the key limiting factors and the real distribution of the species are often unknown (Jiménez‐Valverde et al., 2013; Synes & Osborne, 2011), and statistical methods alone are not sufficient to accept or reject predictors from the ensemble due to conflict rankings (Porfirio et al., 2014). Third, in some regions, the interactive variables that combine information on temperature and precipitation exhibit large shifts in space and time and are therefore often excluded from analyses (Escobar et al., 2014). However, these variables are the most important predictors in some studies (Booth, 2022). Fourth, species distributions often depend more on extremes than on annual means, and extremes are underrepresented in the BCV dataset (Bradie & Leung, 2017; Stewart et al., 2021). Thus, many studies combine BCVs with other (bio)climatic variables that represent extremes.

All these points are controversial and are discussed in detail in recent literature. Only the preselection by expert knowledge based on species limits represents a general agreement (e.g., Porfirio et al., 2014; Synes & Osborne, 2011; Title & Bemmels, 2018). However, two options are commonly used in the absence of general information on the limits. Some studies recommend using the complete set of 19 BCVs, as highly parameterized models with multiple climate predictors may even outweigh possible collinearity problems (Braunisch et al., 2013). In addition, the number of predictors depends on the underlying method that is applied to the data. For example, the BIOCLIM model requires a larger number of predictors in order to achieve similar results as MAXENT with only a limited number of predictors (Booth, 2018; Penman et al., 2010). Other studies consider preselection to be essential since an increasing number of predictors leads to a decrease in the predicted area of suitability (Beaumont et al., 2005) or only improves assessments of the current distribution but performs poorly for projections (Booth, 2018). Porfirio et al. (2014) suggest that preselection is more important for mobile species than long‐lived immobile plants.

While the above issues are mostly taken into account for the use or processing of BCVs in the model‐building phase, the fact that there are multiple ways to calculate BCVs is mostly not considered. To our knowledge, most SDM researchers are not aware of this issue and, therefore, ignore the problems caused by the different calculation approaches. However, there are several calculation schemes that correspond to the definition of BCVs, and different databases are based on different calculation methods, making the results incomparable. The same problem arises with respect to the computational algorithms designed to calculate BCVs, which are available in various computing environments (Bede‐Fazekas & Somodi, 2020). Overall, we found two studies that address different ways of calculating BCVs. O'Donnell and Ignizio (2012) calculated 20 bioclimatic indices for the United States mainly based on the original set of BCVs, but without a comparative analysis of different calculation schemes. In contrast to many other studies, they describe their calculation scheme in detail and list the differences compared to the BCVs of the most popular database, WorldClim. Bede‐Fazekas and Somodi (2020) demonstrate different calculation options in detail, focusing on the temporal context of BCVs. They show that different temporal references, in combination with predictor selection, notably affect model structure and projections. The authors projected changes in potential natural vegetation at high spatial resolution for Hungary using different calculation schemes and regional climate models (RCMs). Depending on the calculation scheme, they projected both large increases and large decreases for some habitats in the future. Therefore, the authors recommend paying more attention to the calculation scheme of BCVs, as it strongly influences the projections. However, both studies did not consider uncertainties arising from different observational datasets. But there are also some studies that deal with intercomparisons of different datasets. For example, Cerasoli et al. (2022) compared the two versions of WorldClim (v1.4, v2.1) for Europe with respect to spatial prediction mismatches, and Morales‐Barbero and Vega‐Álvarez (2019) analyzed mean annual temperatures and precipitation on a global scale using five different datasets to determine bioclimatic congruence. However, to our knowledge, a detailed comparison of the most common gridded station‐based observations and reanalysis weather data for the Mediterranean area and Central Europe with respect to the full set of BCVs has never been performed before.

In addition to the consideration of the effect of different BCV calculation schemes, as recommended by Bede‐Fazekas and Somodi (2020), this study demonstrates how different datasets affect the derivation of BCVs for the extended Mediterranean area. Our results are compared with the most commonly used BCV dataset of WorldClim and the R computational algorithm BIOVARS of the dismo R package. The aim of the present study is to investigate the differences in some BCVs depending on the computational scheme or dataset. We will show that the curse of BCVs does not start during model setup, but already during variable generation.

The data used in this study and the preprocessing steps are described in Section 2.1. For the analysis, we have defined a climatological and biological calculation scheme to identify the wettest, driest, hottest, and coldest month/quarter, which is roughly equivalent to the static and dynamic approach described in Bede‐Fazekas and Somodi (2020). Furthermore, we show different approaches to define, for example, the annual mean temperature (BCV‐1) or the maximum (minimum) temperature of the hottest (coldest) month (Section 2.2). The results are then compared with the BCVs of the WorldClim dataset and with the results of BIOVARS. The differences between the calculation schemes and the datasets among different subregions are shown in Section 3. We discuss the results in Section 4 and draw conclusions in Section 5.

## 2 MATERIALS AND METHODS

### 2.1 Data

#### 2.1.1 Reference

As a reference, we downloaded the set of 19 BCVs from version 2.1 of WorldClim (Fick & Hijmans, 2017) with a spatial resolution of 5 arc minutes, as this is approximately the spatial resolution of our reference dataset in our target domain. The WorldClim dataset contains the climatological means of the 19 standard BCVs for the period 1970–2000. The WorldClim dataset was then interpolated by means of the first‐order conservative remapping algorithm of the Climate Data Operator (CDO; Schulzweida, 2022) to a 0.1° ×  0.1° grid (~90 km^2^ per grid box) to fit the reference spatial resolution over the extended Mediterranean region (10W‐45E, 27N‐55N, 154,000 grid boxes, see Figure 2). In total, we obtain one data value for each BCV of all land grid boxes (113,443 grid boxes).

The WorldClim dataset (https://www.worldclim.org/) can provide a high spatial resolution (30 arc seconds) for the reference period (the future period provides lower spatial resolution), but the temporal resolution (climatological means) as well as the coverage (period 1970–2000) of the BCVs are low. The dataset is limited to land areas but covers the entire world except for Antarctica. In addition, WorldClim provides monthly climatological data for the period 1960–2018 for all variables needed to calculate the BCVs, as well as some other variables such as solar radiation, wind speed, or water vapor pressure (downscaled from CRU‐TS‐4.03). For future projections, they also provide the variables to calculate BCVs based on different shared socio‐economic pathways (SSPs) of 23 General Circulation Models (GCMs). In the following, the original interpolated BCVs of WorldClim are marked WorldClim.

#### 2.1.2 Reanalysis and station‐based gridded datasets

The analyses are based on the ERA5‐Land reanalysis dataset with hourly temporal resolution and 0.1° × 0.1° spatial resolution (Muñoz Sabater, 2019, 2021). Here, the spatial resolution of the ERA5‐Land dataset represents the reference grid for the target domain, and daily or monthly values were derived from the hourly data. ERA5‐Land provides global coverage for the period 1950 to the present. The dataset provides only one spatial resolution, but the temporal resolution ranges from monthly to hourly. In addition to the variables needed for calculating BCVs, more than 50 variables classified into eight groups (e.g., temperature, soil water, radiation, and heat) are available. In addition, different datasets and variables are available for 37 atmospheric levels, which are freely accessible from the Copernicus homepage. Thus, ERA5‐Land represents the most recent dataset with the highest temporal resolution and the largest number of variables in our study domain.

We also consider the gridded Climate Research Unit daily time series (TS) version 4.05 on a regular 0.5° grid (CRU; Harris et al., 2020). The CRU dataset is based on a broad network of weather station observations, and the data are derived by interpolation of monthly anomalies. The dataset provides the coarsest resolution (0.5° × 0.5°) of all datasets considered and covers the period 1901–2020. The dataset provides monthly values of all variables needed to calculate BCVs, plus six additional variables (e.g., vapor pressure, cloud cover; Harris et al., 2020).

Temperature and precipitation time series of the ensembles of the daily gridded observational dataset for precipitation, temperature, and sea level pressure in Europe (E‐OBS, version 24.0e) were downloaded with a spatial resolution of 0.1° × 0.1° (Cornes et al., 2018). The E‐OBS dataset is available in two spatial resolutions (highest resolution: 0.1° × 0.1°) and covers the area from 25° W–45° E and 25° N–71.5° N. Thus, E‐OBS is the only non‐global dataset considered in this study. The dataset provides daily data from 1950 to the end of June 2021. In addition to the variables needed to calculate BCVs, four other variables are available (e.g., sea level pressure, relative humidity; Cornes et al., 2018). As E‐OBS is only based on observations, the dataset contains gaps, and thus some regions, periods, and/or variables are not continuously represented. Generally, a grid box within the E‐OBS dataset is considered complete when 80% of the data is available.

All datasets have been fitted to the grid of the target domain and interpolated where necessary by means of the same interpolation scheme applied to WorldClim. Since all datasets are derived by different methods, in the following, we summarize ERA5‐Land, CRU, and E‐OBS under the term gridded station‐based observations and reanalysis weather data (GSORW‐Data). Although WorldClim is also a gridded station‐based observational dataset, it only represents climatological means instead of daily meteorological data. For the comparative analysis between WorldClim and GSORW‐Data, we consider the period 1970–2000, since the BCVs of WorldClim only cover this period. For intra‐ and inter‐comparisons of the GSORW‐Data, we extend the period to 1970–2020. We downloaded hourly, daily, or monthly values of minimum, mean, and maximum temperature as well as precipitation sums for all datasets and aggregated them to monthly values using CDO where appropriate. All analyses and figures were performed using R Statistical Software (v4.2.2; R Core Team, 2022) and RStudio (Posit Team, 2022).

### 2.2 Calculation schemes

There is not much information in the literature on the calculation of WorldClim BCVs. Several approaches are possible to derive the 19 BCVs from temperature (T2M) and precipitation (PRE) time series. In particular, the definition of the hottest/coldest or wettest/driest quarter/month provides some options (Bede‐Fazekas & Somodi, 2020; O'Donnell & Ignizio, 2012). Following BIOVARS provided by the creators of the WorldClim dataset, we assume that all BCVs of WorldClim are calculated using climatological means over a period of 31 years (1970–2000). Thus, each grid box is not represented by a time series but by monthly climatological mean values for each climate variable (i.e., minimum and maximum temperature, precipitation).

The WorldClim annual mean temperature (TMEAN) is the average of the maximum and minimum temperatures. Since most of the popular reanalysis and observational datasets (e.g., ERA5(‐Land), CRU, E‐OBS, and NCEP‐NCAR) provide mean temperatures, the calculation of the annual mean temperature is redundant, but since we have also applied BIOVARS, the calculation of the mean using the maximum (TMAX) and minimum (TMIN) temperatures is considered too. Differences within the calculation scheme also affect BCV‐4.

Several options are available for BCVs defined for specific periods of interest (POI, month, or quarter). When climatological averages (e.g., over a 30‐year period) are used as input, the period with the highest or lowest average values will always represent the POI. However, if time series are used, the POI can be defined in two ways. First, we define the POI for the entire time series, that is, using the driest month on average in the time series. Then we extract the corresponding month from all years and calculate the average, even though the month is not the driest in every year of the time series (climatological approach, CLIM). On the other hand, the POI is identified separately for each year, and then the respective values are averaged. In this way, the driest month of each year is always taken into account, although it is not usually the same month throughout the period (biological approach, BIO). Such a concept is also pursued by Bede‐Fazekas and Somodi (2020), but for the dynamic or static selection of quarters/months with respect to future periods. However, their method is also valid for and applicable to recent periods in order to calculate the BCVs, and Bede‐Fazekas and Somodi (2020) also provide similar information in the Appendix S2. The climatological approach is consistent over time since the BCV always represents the same month or quarter of the year, but due to interannual shifts, the definition of the BCV is no longer valid. On the other hand, the biological approach is always consistent with the definition of BCV, since it always represents the month or quarter with the highest or lowest value but describes different physiological and ecological phenomena, for example, when the driest month is shifted from the early growing season to the hibernation season. Thus, the statistical link established during model construction is questioned (Bede‐Fazekas & Somodi, 2020). This problem concerns all BCVs related to specific months (BCV‐5, BCV‐6, BCV‐13, BCV‐14, and indirectly BCV‐3 and BCV‐7) or quarters (BCV‐8–BCV‐11 and BCV‐16–BCV‐19).

For BCV‐5 and BCV‐6, there are two ways to extract the extreme monthly temperatures. On the one hand, the absolute lowest/highest daily value of the month can represent the minimum/maximum temperature of the coldest/hottest month. On the other hand, the BCVs can be calculated using the averaged minimum/maximum daily temperatures of the respective month. The same method has also been applied to periods or annual time series to show the effect of the calculation methods on different time scales (e.g., the lowest daily temperature of the year vs. the average of the daily minimum temperatures of the year). These two options indirectly affect the calculation of BCV‐2, BCV‐3, and BCV‐7 as well. In the following, we show the difference between the two calculation schemes (see Section 3.1).

An additional aspect must be considered when the BCVs are based on quarters (three consecutive months). When BIOVARS is applied to annual data, the quarter (November–December–January) is calculated using the monthly data of the same year. Thus, January of the same year is added to November and December, even though they are not consecutive. This calculation scheme may be valid (but also inaccurate) for climatological averages but not for time series (O'Donnell & Ignizio, 2012). The effect on temperature‐based quarters of time series may be marginal, as the variability of monthly mean temperatures is relatively small compared with the dominant seasonal cycle, but for precipitation‐based quarters, the differences can be substantial. Another issue is the definition of quarters, that is, whether the first quarter starts with January or whether January is the month in the middle. If January is the middle month, it is recommended to extend the time series of interest by 2 years (start year −1):(end year +1) in order to calculate all quarters by consecutive months; otherwise, 1 year should be added at the end of the time series. This feature concerns all BCVs based on quarterly values (BCV‐8‐BCV‐11, BCV‐16‐BCV‐19). However, we do not address the variations in the calculation of BCVs that result from these issues. In this study, we calculate BCVs based on consecutive months, using the median month as a representative.

We compared different datasets (ERA5‐Land, E‐OBS, and CRU) based on time series (Section 3.2). The BCVs of the time series were extracted using BIOVARS as well as the BIO and CLIM calculation schemes. In the following, the quarters of the BIO and CLIM calculation scheme are based on consecutive months, and the reference is always the month in the middle. Thus, the first quarter of 1970 includes the months December 1969–February 1970.

## 3 RESULTS

In the following, we first present the differences between different calculation schemes using the ERA5‐Land reanalysis dataset for selected BCVs. Results are exemplified for the annual mean temperature (BCV‐1) to show differences that occur when using mean temperature time series or mean values derived from minimum and maximum temperature. Differences between absolute and mean extreme temperatures are illustrated by means of the maximum temperature of the warmest month (BCV‐5). Differences arising from the CLIM and BIO calculation schemes are presented by means of the mean temperature and precipitation of the wettest quarter (BCV‐8 and BCV‐16). An intercomparison of different datasets is presented in Section 3.2. Since all BCVs are based on temperature and precipitation and mean annual values of climate variables are a good indicator of how well these are represented by different datasets, we show the results for the annual mean temperature (BCV‐1) and precipitation (BCV‐12). To show whether the selection of the quarters is represented differently within the GSORW‐Data, results are also visualized for the mean temperature of the wettest quarter (BCV‐8). Figures for other monthly or seasonal BCVs affected by the aforementioned differences in calculation schemes are presented in Appendix S1 and differences with respect to different datasets are presented in Appendix S2.

### 3.1 Differences of BCVs due to calculation scheme

**FIGURE 1:**
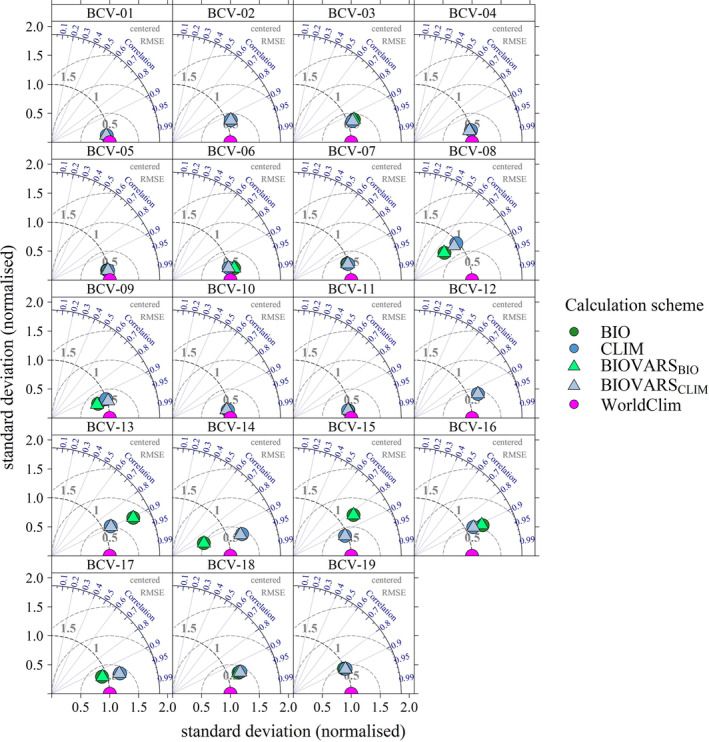
Taylor‐Diagrams depicting the differences with respect to the calculation scheme for all BCVs (Period: 1970–2000; Reference: WorldClim). Due to different scales, the standard deviations are normalized and the RMSEs are centered. The points represent the biological (BIO, green) and climatological (CLIM, blue) calculation schemes, and triangles represent the BCVs derived from BIOVARS when the function is applied to time series (BIOVARS_BIO_, light green) or periods (BIOVARS_CLIM_, light blue).

Figure 1 shows Taylor plots (Taylor, 2001) for all BCVs derived from the ERA5‐Land dataset. The Taylor plots show the normalized standard deviation, the centered root mean square error (RMSE), and the correlation coefficient of the different calculation schemes with respect to the reference BCVs of WorldClim (purple point in the middle of the *x*‐axis). In general, the closer the data point is to the reference, the higher the agreement between the reference and the calculation scheme. The BCVs of WorldClim are used as a reference. The Taylor plots show only minor differences between the calculation schemes when only temperature variables are involved. The differences increase when precipitation values are considered and show considerable variations when the POI is determined by precipitation, that is, wettest month (BCV‐13, BCV‐14) or wettest quarter (BCV‐8, BCV‐9, BCV‐16, BCV‐17). The figure also shows that the BIO approach is similar to the derived results of BIOVARS_BIO_, and CLIM approximately corresponds to BIOVARS_CLIM_. Thus, notable differences are only due to whether the BCVs are calculated from climatological means (CLIM) or time series (BIO), whereas the differences resulting from the calculation scheme and BIOVARS are only marginal.

#### 3.1.1 Annual mean temperature (BCV‐1)

**FIGURE 2:**
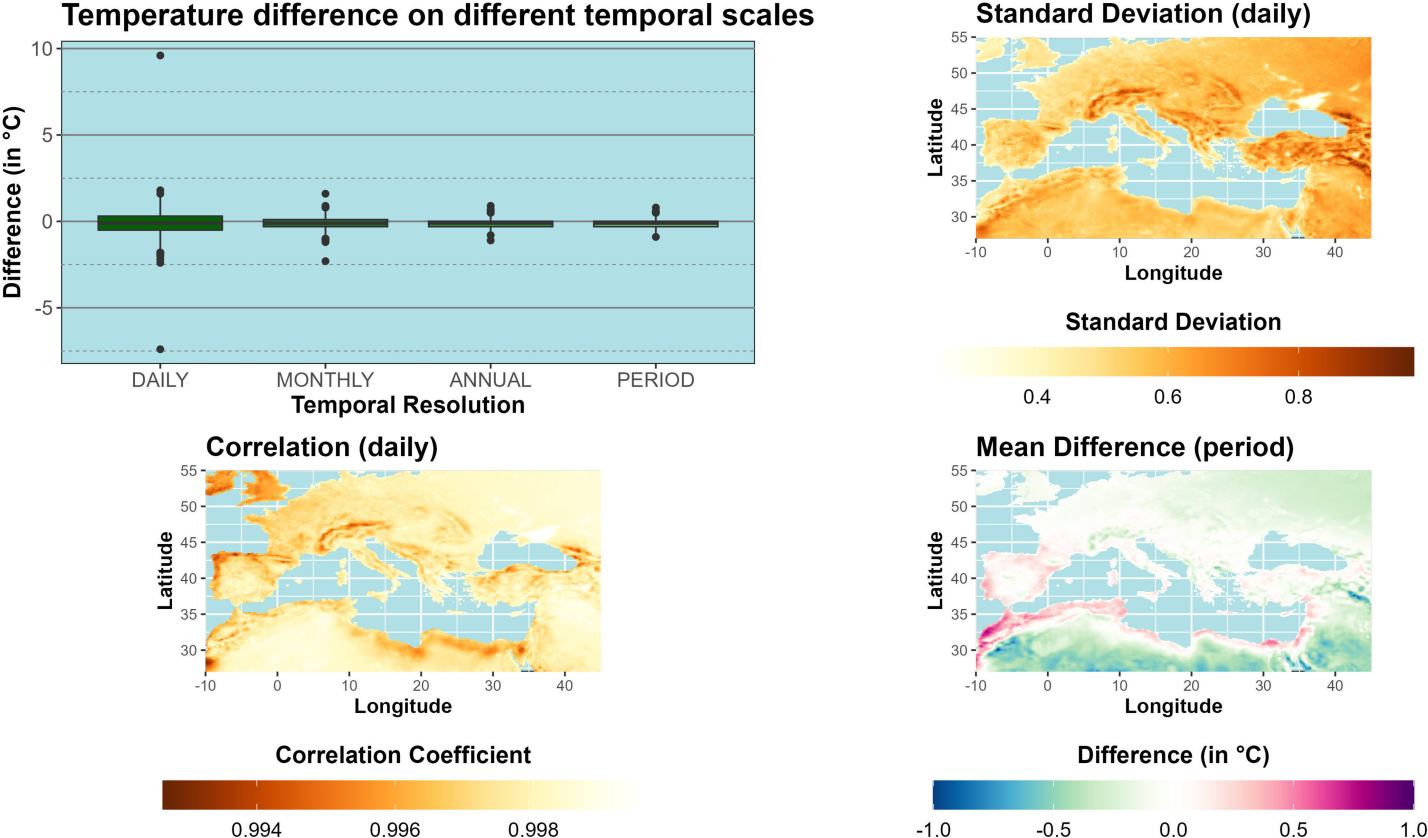
BCV‐1 Annual mean temperatures: Differences between WorldClim's calculation scheme and mean temperature time series of ERA5‐Land for different temporal scales (top, left), standard deviations (top, right), and correlation coefficient (bottom, left) of daily temperatures. Mean differences between both time series are shown for the period 1970–2020 at the bottom, right (BIOVARS–ERA5‐Land).

Differences with respect to the BCV‐1 calculation scheme are shown for ERA5‐Land in Figure 2. The difference between the TMEAN of ERA5‐Land and the calculation using minimum and maximum temperatures depends on the temporal resolution. On a daily scale, differences between −7.4°C and +9.6°C occur and become smaller when the temporal resolution is reduced. If only the range between the lower and upper quartile is considered, the monthly, annual, and interannual time series are almost identical, unless the range of the daily quartile is extended by 0.2°C in both directions. For a period of 31 years, the deviations between the two approaches do not exceed ±1°C (top left). Especially in mountainous regions (e.g., Alps, Atlas, and Caucasus), the standard deviations of the differences show higher and the correlation coefficients lower values. Although the standard deviations within these regions are quite low, lower correlation coefficients also occur on the Sinai Peninsula, the northwestern coasts of the Iberian Peninsula, and the southern coast of Ireland (bottom left). The highest agreement between the two methods is found over the Sea of Azov. Overall, ERA5‐Land TMEAN exceeds the temperature time series derived from minimum and maximum temperatures for most parts of the study area, with the highest values in the northern parts of Africa and the Middle East away from the coast. Along the southern and eastern coasts of the Mediterranean Sea and over the Iberian Peninsula, the TMEAN of ERA5‐Land is notably lower than that computed by BIOVARS, with the largest deviations along the Atlantic coast of Morocco.

#### 3.1.2 Maximum temperatures of the warmest month (BCV‐5)

**FIGURE 3:**
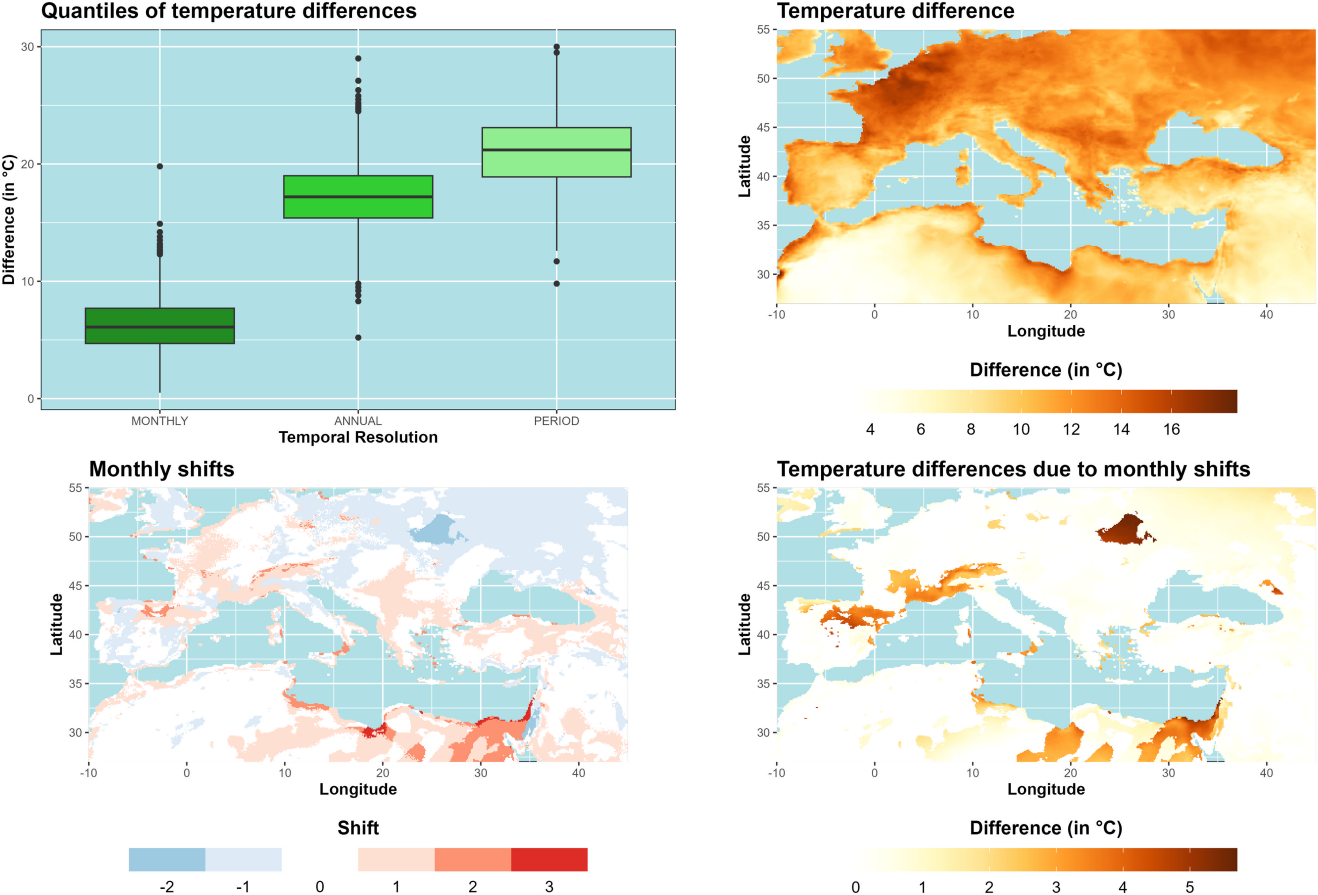
BCV‐5 Maximum temperatures of the warmest month: Differences between absolute and mean maximum temperatures (period 1970–2020) of ERA5‐Land for different temporal scales (top, left) and regions (top right). The temporal shift (in months) of absolute and mean maximum temperature is presented at the bottom left. The shift‐induced temperature difference of the mean maximum temperature is given at the bottom right.

Absolute and mean maximum/minimum temperatures were calculated for the period 1970–2020 on a monthly and annual basis, as well as for the entire period, by means of the CLIM approach. In contrast to BCV‐1, the temperature differences become larger with reduced temporal resolution (see Figures 2 and 3, upper left), but the differences are already notable for monthly time series. Here we present the results for the maximum temperature of the warmest month (Figure 3). Especially in Northern France and the Benelux countries, temperature differences of more than 15°C can be observed between the two calculation schemes (Figure 3, top right). Since mean and absolute maximum temperatures do not always occur within the same month, both calculation schemes are also subject to temporal shifts. On average, the absolute maximum temperature at the Israeli‐Egyptian coast is 3 months later than the mean maximum temperature, while the absolute values reach their maximum 2 months earlier than the mean temperatures in the northwestern parts of Ukraine (Figure 3, bottom left). Finally, we used the date of the maximum from the absolute and mean maximum (derived from the entire period) and extracted only the mean maximum temperatures for both time series. Considering only the temporal shift between absolute and mean maximum temperatures, the shift of the maximum alone leads to differences of up to 5.7°C in some regions (Figure 3 bottom right, Border area of Ukraine and Belarus, Mediterranean coast of Israel and Lebanon).

#### 3.1.3 Mean temperature/precipitation of the wettest quarter (BCV‐8, BCV‐16)

**FIGURE 4:**
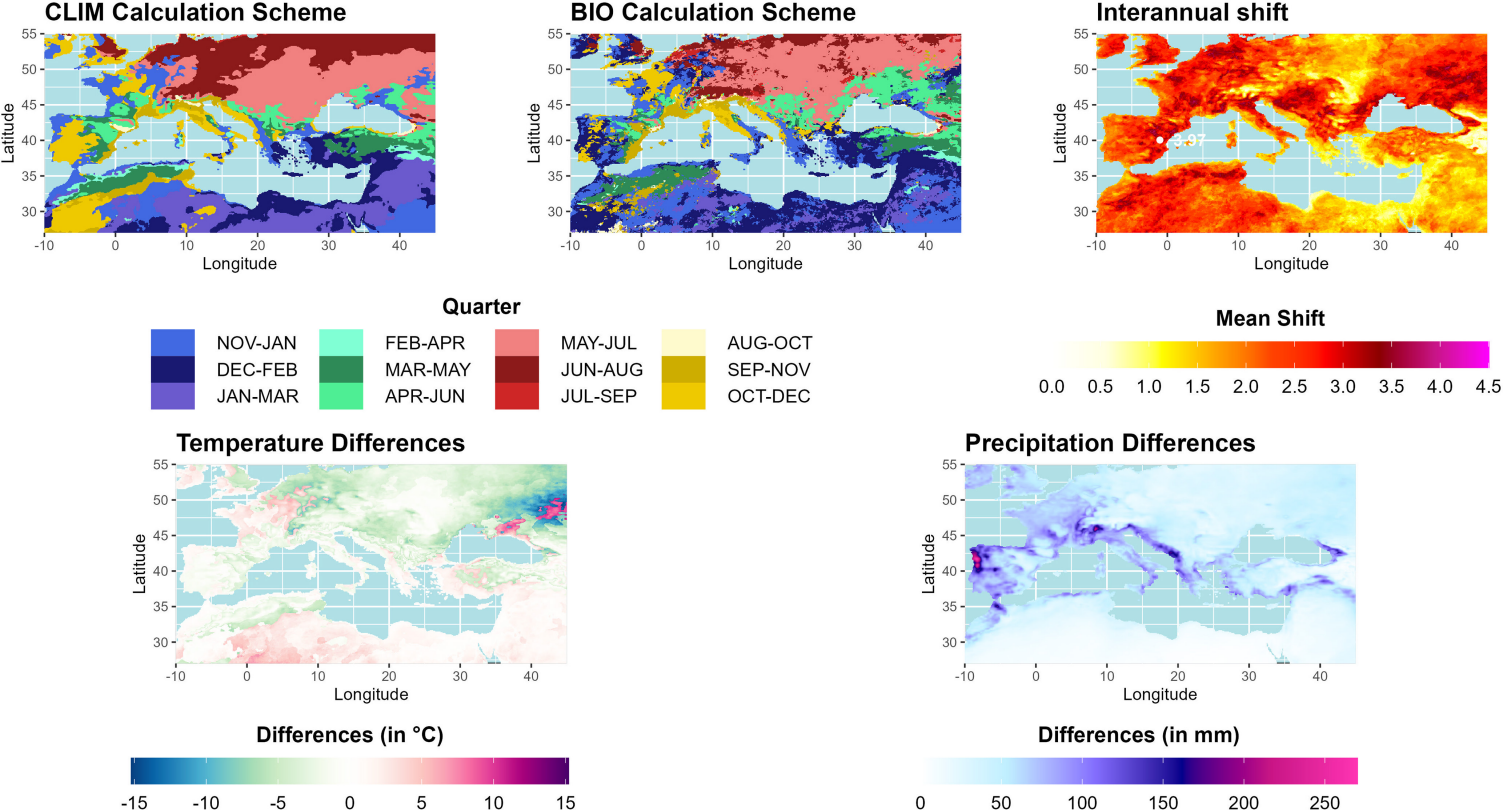
BCV‐8 and BCV‐16 Temperature and precipitation in the wettest quarter: The upper figures represent the climatological quarters of the wettest quarter (CLIM, top left) and the quarter that most frequently represents the wettest quarter due to the biological approach (BIO, top mid) of the ERA5‐Land dataset (period 1970–2020). The figure at top right represents the mean interannual shift of the wettest quarter for the BIO calculation scheme (Maximum shift: white dot and number). Figures at the bottom show the differences between both approaches (BIOCLIM) with respect to temperature (left) and precipitation (right).

A rough classification of the study area highlights four subregions with respect to the climatologically wettest quarter (Figure 4). Beginning with the southern subregion in winter, the date of the wettest quarter shifts counterclockwise through the seasons. The Black Sea region has the wettest quarter in spring, the northern subregion in summer, and the western parts of the Mediterranean region in autumn (Figure 4, upper left). However, a closer look at the Iberian Peninsula shows that although the climatological wettest quarter is in autumn, the winter season quantitatively represents the wettest quarter (BIO calculation scheme, Figure 4, upper middle). Overall, the BIO approach shows a more heterogeneous picture, with shifts mainly within the respective season. In the upper right part of Figure 4, the interannual shifts of the wettest season are shown (BIO calculation scheme). It shows that the wettest season is shifted by up to 4 months per year on average north and south of the Pyrenees, northeast of the Black Sea, and in parts of Bulgaria. This reflects the high interannual variability of rainfall in the extended Mediterranean area. Smaller interannual shifts occur in the southeastern parts of our study area, in the eastern parts of Europe, and north of the Alps. In terms of temperature, these shifts have the greatest impact northeast of the Black Sea, with high gradients between −10°C and +10°C, while the northwestern parts of the Iberian Peninsula show the greatest differences in precipitation (−250 mm).

### 3.2 Differences of BCVs due to datasets

**FIGURE 5:**
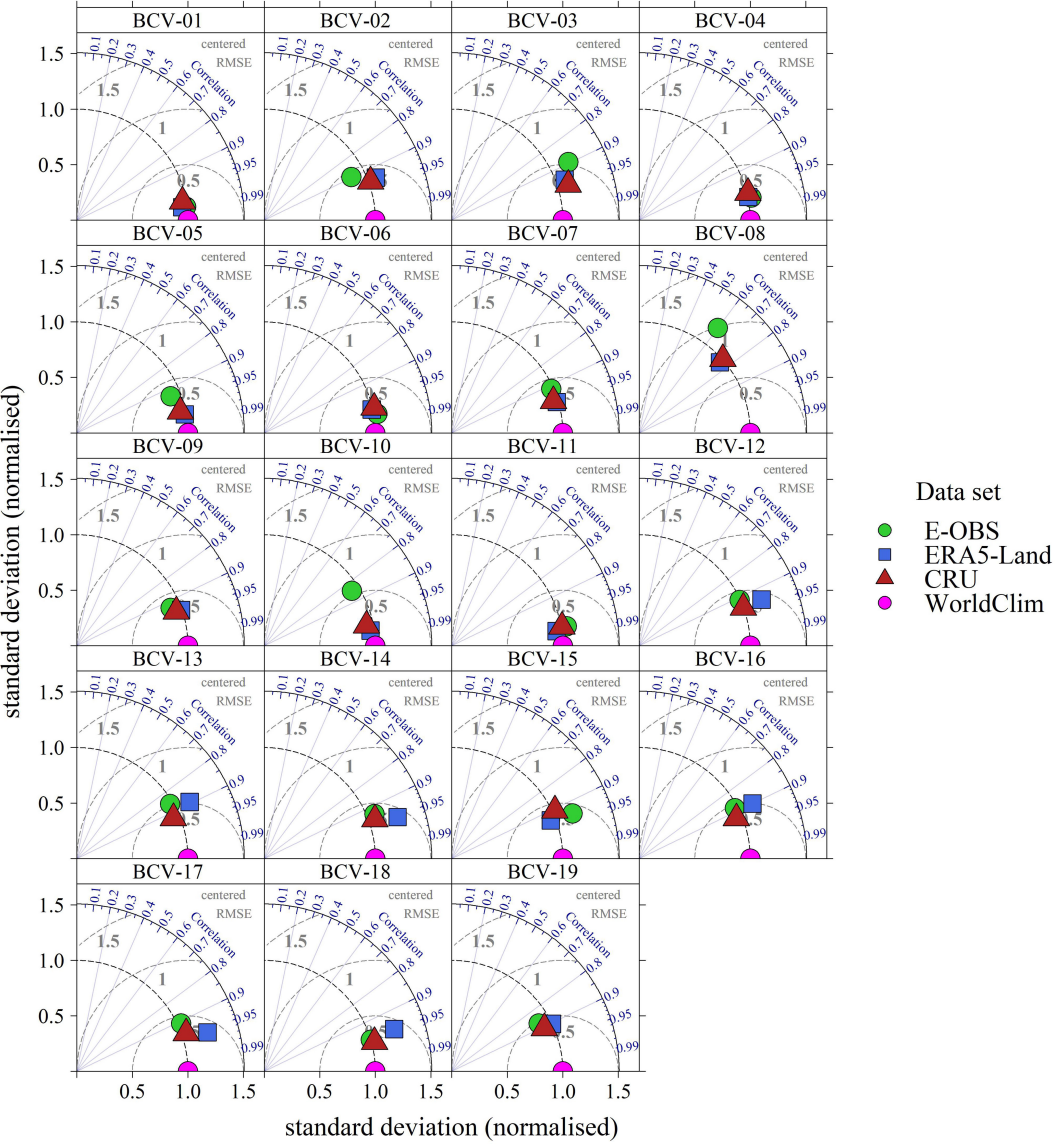
Taylor‐Diagrams depict differences with respect to the GSORW‐Datasets for all BCVs (Period: 1970–2000; Reference: WorldClim). Due to different scales, the standard deviations are normalized and the RMSEs are centered.

In Figure 5, Taylor plots are shown for all BCVs derived from different datasets using the CLIM calculation scheme as a reference. Otherwise, the framework is the same as in Figure 1. The Taylor plots show that all datasets reproduce the BCVs of the WorldClim database well, and major differences between the models can only be observed for BCV‐8 and BCV‐10 of temperature and, to some extent, BCV‐13 of precipitation. On the one hand, possible reasons for the deviations can be traced back to the different representation of precipitation within the GSORW‐Datasets, which affects precipitation‐based BCVs (e.g., BCV‐13) as well as temperature‐based BCVs (e.g., BCV‐8). When regions have two precipitation peaks with similar amounts in summer and winter, temperatures can vary notably, while the precipitation of the wettest quarter (BCV‐16) shows only small variations. In addition, precipitation has higher spatial and temporal variability, so different resolutions and interpolation schemes may also affect the representation of precipitation patterns. For BCV‐10, larger deviations are observed only in E‐OBS. Here, spatial and temporal data gaps may be the reason for the deviations since the temperature is generally well represented among the GSORW‐Datasets.

In the following, WorldClim is used as a reference for all other datasets. When we use evaluative terms, we are referring to the WorldClim dataset without claiming that this dataset is the best representation of reality.

#### 3.2.1 Annual mean temperature (BCV‐1)

**FIGURE 6:**
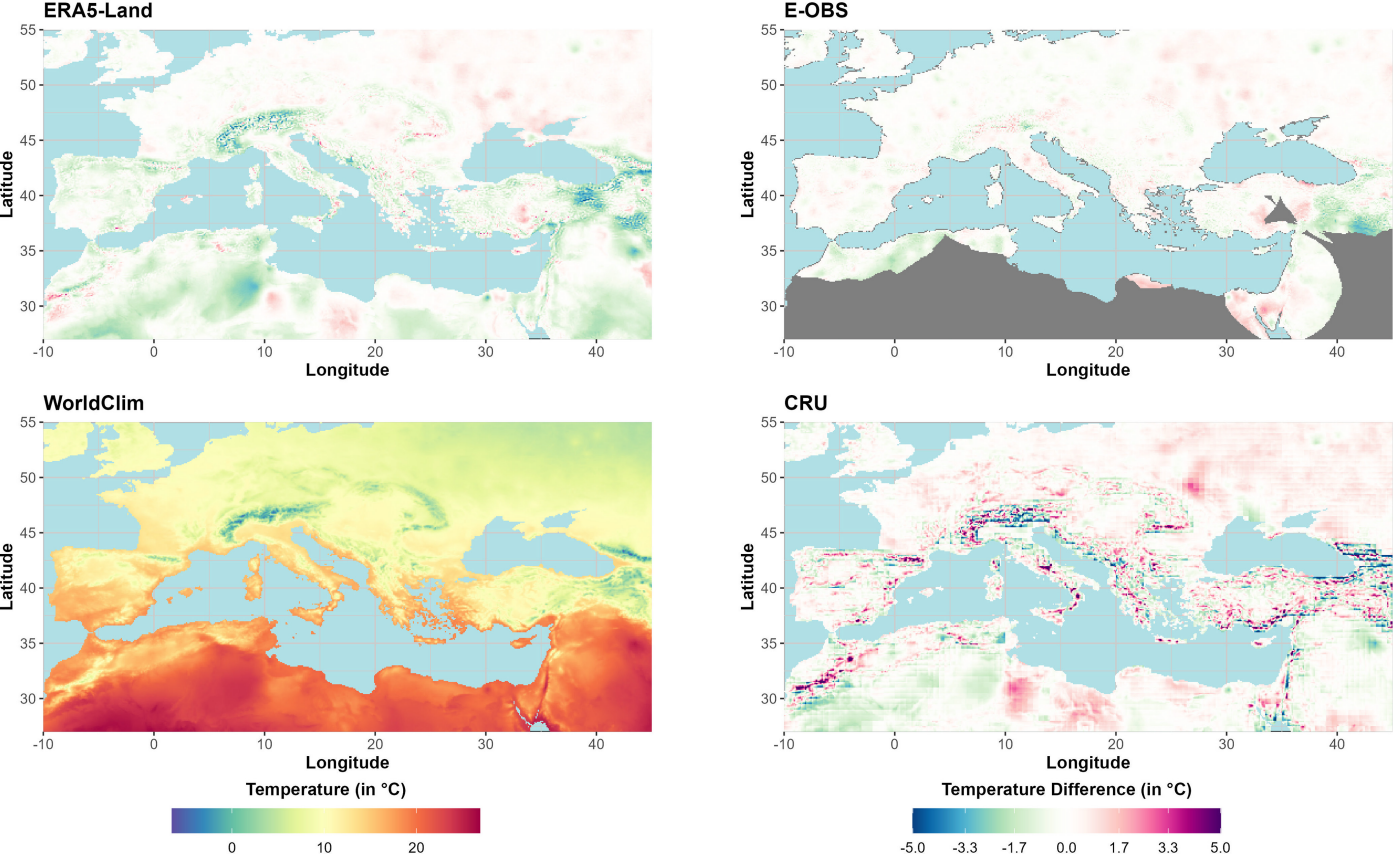
BCV‐1 Annual mean temperature: The figure shows the temperatures of WorldClim (bottom left) and the temperature differences (dataset minus WorldClim) of ERA5‐Land (top left), E‐OBS (top right), and CRU (bottom right) for the period 1970–2000 (gray areas: no data).

Figure 6 shows the annual mean temperatures of WorldClim (BCV1) for the period 1970–2000 and the temperature anomalies of the different GSORW‐Datasets. Compared to the other datasets, E‐OBS shows the smallest deviations (−3.4 to 2.6°C). Especially in the eastern parts of Turkey, the annual mean temperatures are far below those of WorldClim. ERA5‐Land shows negative deviations over much of North Africa but also over Turkey and the Levant, Spain, and the Alpine region, and the deviations are higher than those observed in E‐OBS (−5.6 to 3.8°C). The highest deviations are observed within the CRU dataset (−10.0 to 9.2°C). Especially in mountainous regions, the CRU dataset exhibits both large negative and positive deviations. Overall, all datasets show higher mean annual temperature differences to WorldClim in the northeastern part of the study area, while lower temperature differences are observed along the southern Mediterranean coast. The latter is probably due to the limited data availability in this region and, thus, a more similar database in all considered datasets.

#### 3.2.2 Mean temperature of the wettest quarter (BCV‐8)

**FIGURE 7:**
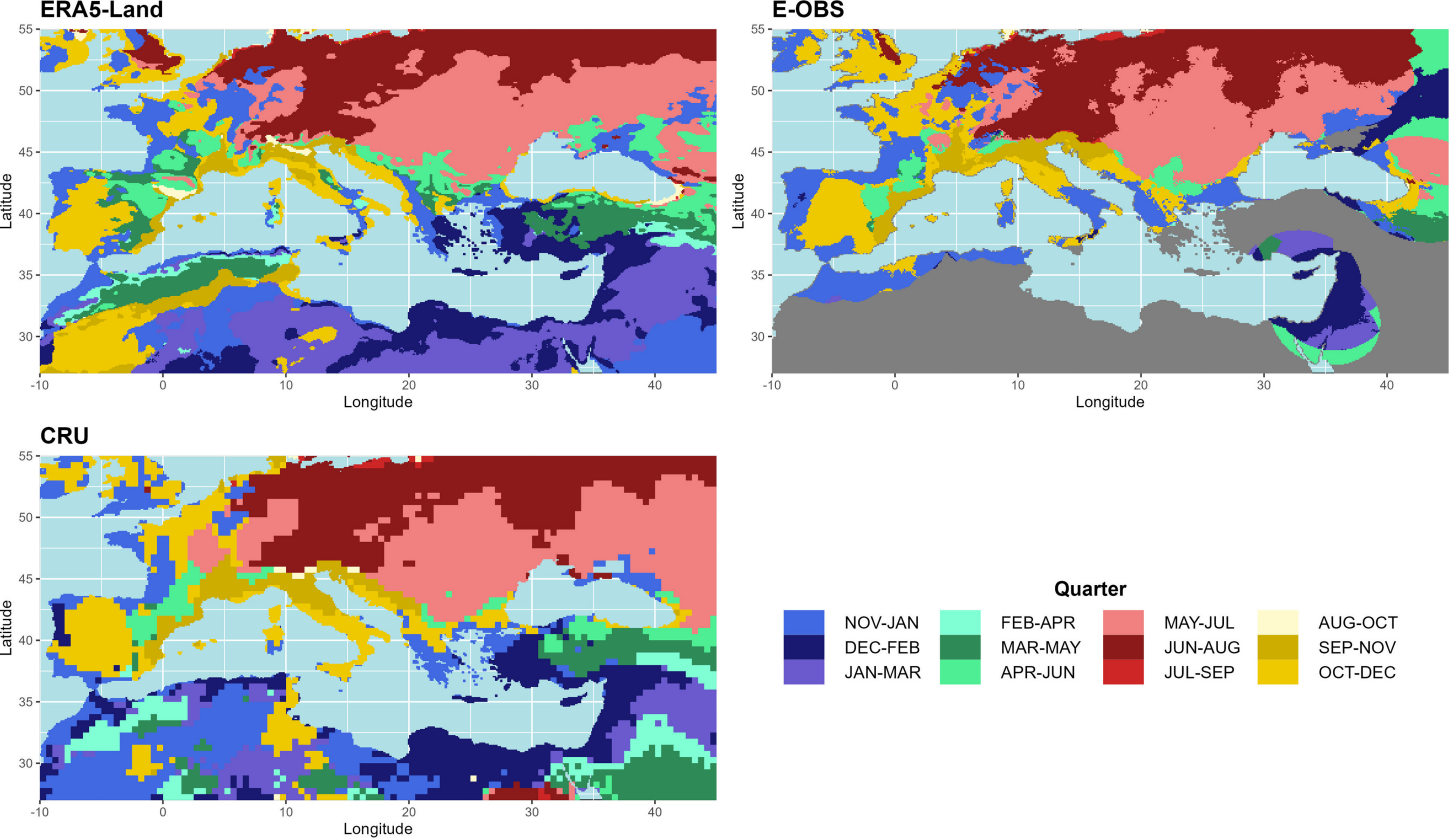
Wettest quarter for ERA5‐Land (top left), E‐OBS (top right), and CRU (bottom left) for the period 1970–2020 (gray areas: no data).

Figure 7 shows that the Mediterranean and European areas are very heterogeneous in each GSORW‐Dataset where the wettest quarter can occur in any season. Thus, even small shifts in the precipitation pattern or deviations in the spatial resolution can lead to notable changes in BCV‐8. The greatest agreement is observed over central and eastern Europe, where precipitation maxima occur in the summer. The largest discrepancies are observed northeast of the Black Sea within the CRU dataset, but large discrepancies are also observed over France, where precipitation maxima occur in all seasons. Over the Iberian Peninsula, the adjacent regions with precipitation maxima in spring and autumn are represented in all datasets, although some shifts in the boundaries can be observed. Over the southeastern parts of the study area, ERA5‐Land shows precipitation maxima in winter, while CRU and, where data are available, E‐OBS show the wettest quarter in spring.

**FIGURE 8:**
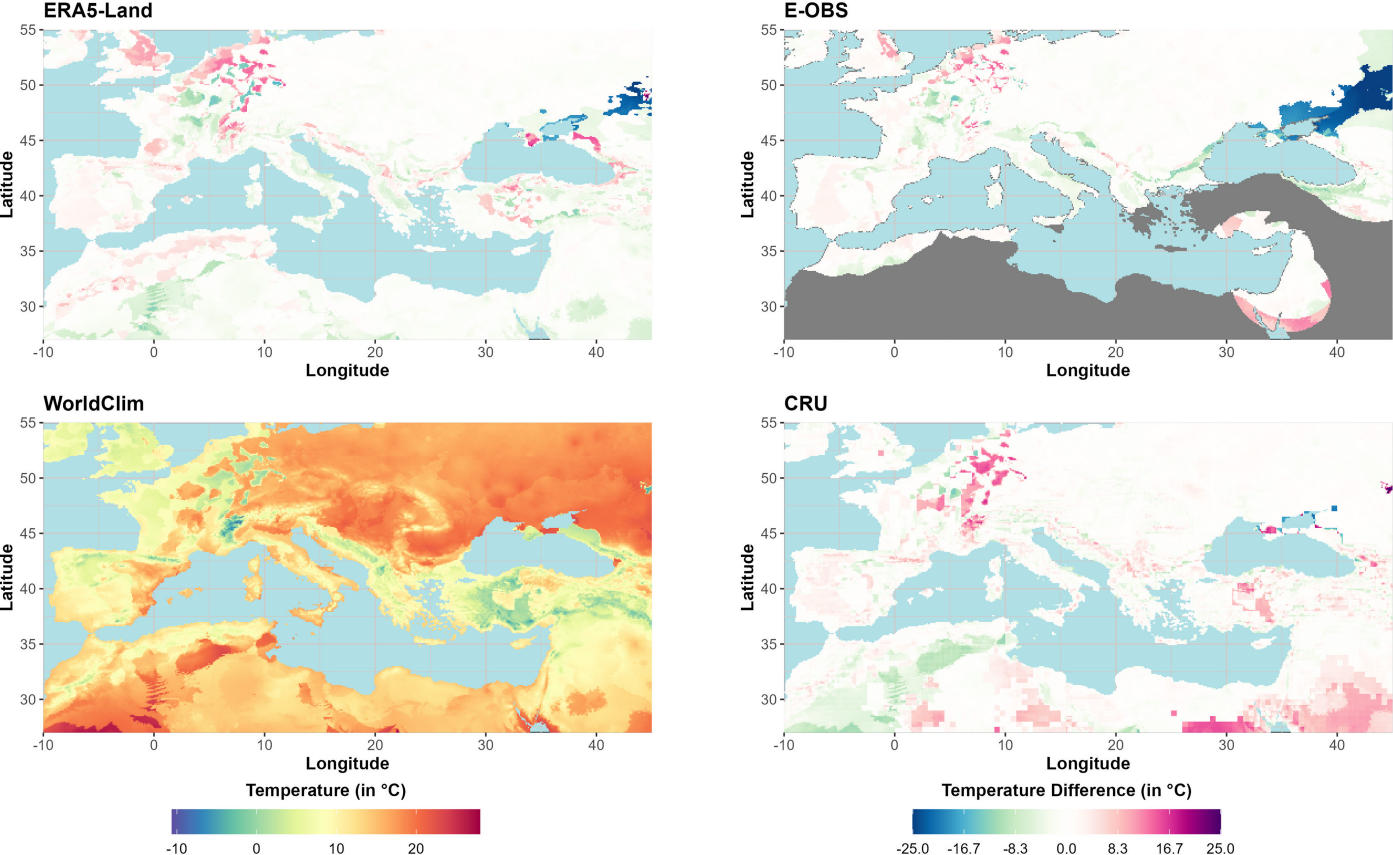
BCV‐8 Mean temperature of the wettest quarter: The figure shows the temperatures of WorldClim (bottom left) and the temperature differences of ERA5‐Land (top left), E‐OBS (top right), and CRU (bottom right) for the period 1970–2000 (gray areas: no data).

The heterogeneity in France is responsible for the large temperature differences within this region. Between the Gulf of Lion and the German North Sea coast, the temperatures of the wettest quarter are for some regions more than 10°C higher in the GSORW‐Data than in WorldClim (Figure 8). Over the northeastern parts of the Black Sea, we assume that WorldClim also has its wettest quarter in summer since CRU almost matches the reference pattern. In contrast, warmer temperatures are observed over the Sinai Peninsula for CRU and, where data are available, for E‐OBS, leading to the conclusion that both WorldClim and ERA5‐Land have their wettest quarter in winter. In general, within the considered domain, the datasets show warmer temperatures than WorldClim, but also notably lower temperatures can be observed.

#### 3.2.3 Annual precipitation (BCV‐12)

**FIGURE 9:**
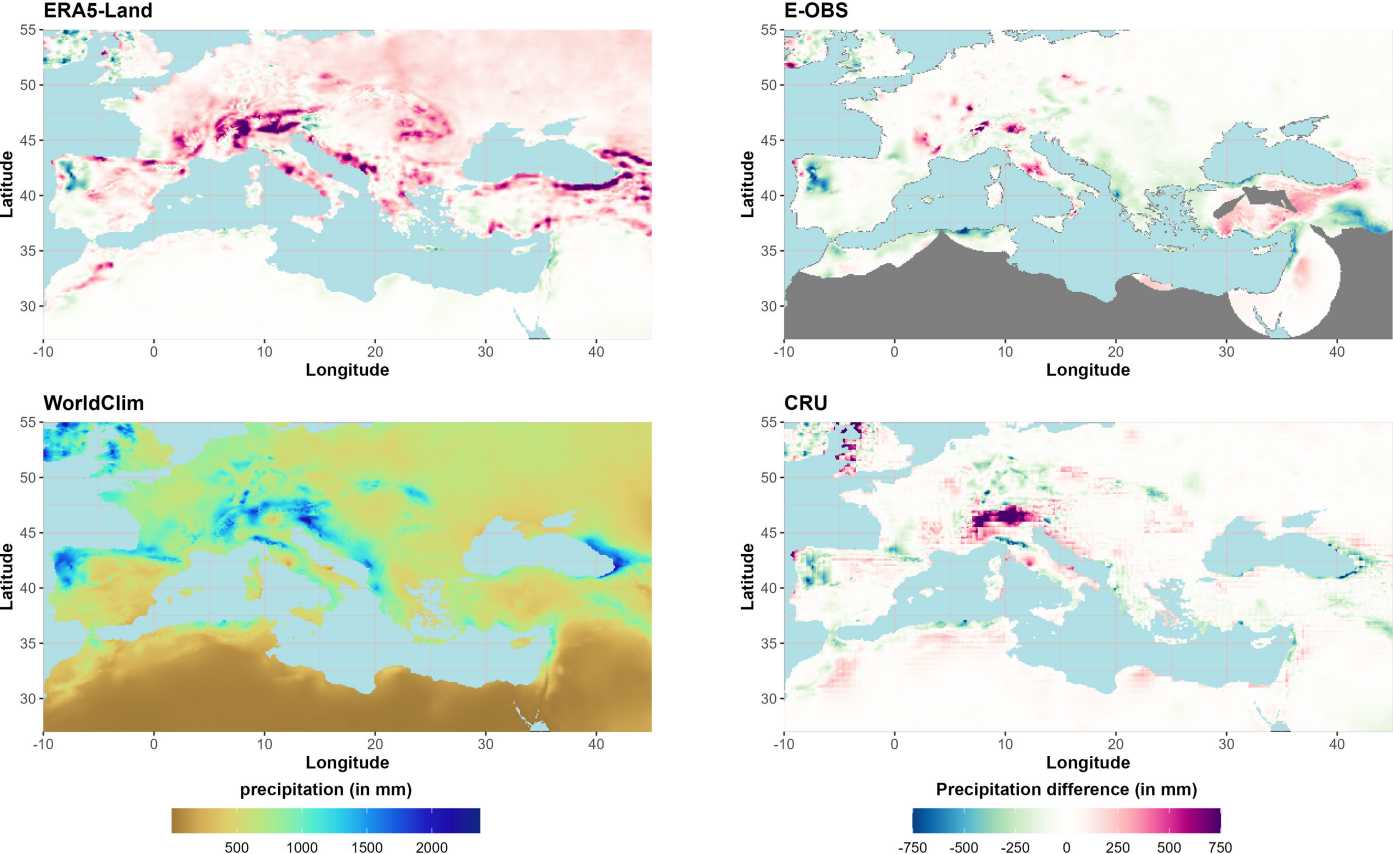
BCV‐12 Annual mean precipitation: The figure shows the precipitation of WorldClim (bottom left) and the precipitation differences of ERA5‐Land (top left), E‐OBS (top right), and CRU (bottom right) for the period 1970–2000 (gray areas: no data).

The largest differences between the datasets and WorldClim are observed in the triangle between France, Switzerland, and Italy on the west side of the Alps (Figure 9). Here, all datasets overestimate the WorldClim annual precipitation totals by more than 1000 mm, with the largest deviations within the ERA5‐Land dataset (2200 mm). Differences between the datasets can be observed at the southeastern coast of the Black Sea, where CRU shows the highest underestimation (−1157 mm). In this area, ERA5‐Land overestimates precipitation by 482 mm and E‐OBS by 109 mm. Compared to WorldClim, ERA5‐Land (+77 mm) and CRU (+2 mm) are too wet, while E‐OBS (−30 mm) is too dry on average. Especially over topographically heterogeneous areas such as the Alps or the eastern Pontic Mountains south of the Black Sea, precipitation amounts are predominantly overestimated, while the western part of Europe (northwest of the Iberian Peninsula, Great Britain, and Ireland) shows lower precipitation amounts than the reference.

## 4 DISCUSSION

### 4.1 Differences between calculation schemes

Analyses have shown that there is a high degree of agreement between all the calculation schemes presented. Only BCVs based on periods (month or quarter) defined by precipitation show larger differences (BCV‐8, BCV‐9, BCV‐13, BCV‐14, BCV‐16, BCV‐17). It has been shown that it does not matter whether the BCVs are calculated with one of the calculation schemes presented here or with BIOVARS provided by the dismo R package, since the differences are only marginal. For the calculation of BCVs, it is only relevant whether the variables are calculated by means of annual values or period means. Thus, although there are small differences between BIOVARS and the calculation schemes, these have hardly any impact on the calculation of the BCVs. Consequently, BIOVARS proves to be a suitable method to obtain BCVs for the Mediterranean region. It should only be thoroughly checked if the wettest or driest season coincides with the turn of the year. With respect to BCV‐1, the higher the temporal resolution, the greater the differences between the mean temperatures provided by the datasets and the calculation scheme of BIOVARS. Thus, only marginal differences will occur when the function is applied to annual or climatological means. However, Hijmans (2006) and Hijmans et al. (2022) explicitly point out that the function can also be applied to weekly data. In our opinion, the user should rather refer to the original mean temperature time series of the used dataset, since, especially over mountainous regions or regions with a high degree of continentality, differences can be large at high temporal resolution, and, thus, the use of BIOVARS for calculating BCV‐1 can lead to notable differences in comparison to the mean temperature time series of the GSORW‐Data. Furthermore, since all datasets provide mean temperature time series, the calculation of mean temperatures is redundant.

For the time series of maximum/minimum temperatures of the warmest/coldest months (BCV‐5 and BCV‐6), the two calculation schemes show large differences when the temporal resolution is reduced. Analyses have shown that the BCVs of WorldClim correspond to the calculation scheme with mean maximum or minimum temperatures. It has been shown that differences of up to 16°C occur between the two calculation schemes, especially in the oceanic regime. Here, the moist air masses of the Atlantic Ocean flatten the daily temperature amplitude, so that single extremes notably increase the difference between absolute and mean maxima. In addition, calculating mean maximum temperatures by considering only the temporal shift between absolute and mean maxima also results in temperature differences of up to 6°C. The largest differences are observed along the Egyptian Mediterranean coast, where shifts of up to 3 months occur between absolute and mean maximum temperatures, but also along the French Mediterranean coast, where the temporal shift is only 1 month. It shows that large shifts between mean and absolute temperature maxima occur mainly along the North African coast, but the effect is similar to small shifts in some European regions. However, we assume that the approach based on mean maxima/minima should be applied when mobile species are investigated or the temperature limits of the species cannot be specified exactly, whereas absolute maxima/minima should be considered when the temperature limits of species are known and essential for survival. Especially for immobile species such as plants, the use of absolute minima and maxima may have added value. This is also consistent with the analysis of Gloning et al. (2013) on the calculation of hardiness zones for woody plants. The authors suggest that absolute minimum temperatures should also be considered as the future climate is warmer, but the risk of cold snaps is still present due to increased standard deviations and changes in the skewness of the minimum temperature distribution. Therefore, the choice of calculation scheme depends strongly on the species and the research question.

The most controversial variables in the BCV pool are the ones that combine temperature and precipitation. Especially large changes within short distances are an issue of major concern. These discontinuities are predominantly related to shifts in the quarterly periods used to calculate the BCVs (Booth, 2022). For example, the mean temperature of the wettest quarter (BCV‐8) can exhibit notable differences in regions with two rainy seasons in summer and winter, and maxima are shifted spatially (one grid box has a maximum in summer and the neighboring grid box in winter) or temporally (summer maximum in 1 year and winter maximum in the following year). Temporal shifts also affect the dynamic approach (similar to the BIO calculation scheme when applied to different climatological periods) described in Bede‐Fazekas and Somodi (2020), where variables are determined period‐by‐period. This leads to a discussion about how to handle these BCVs. On the one hand, Escobar et al. (2014) pointed out that these variables should be excluded from SDM since they show odd spatial anomalies in some regions. Authors who cite Escobar et al. (2014) even claim that these variables are unsuitable for usage (Booth, 2022). On the other hand, Bradie and Leung (2017) have analyzed different environmental variables and their contribution to SDM. They concluded that the interactive variables are the most important predictors in some regions and for some species. For global studies, Booth (2022) recommends seeking alternative measures for interactive variables based on water balance studies, as rainfall seasonality can be important for species distributions.

In addition to the question of the usefulness of the interactive variables, Bede‐Fazekas and Somodi (2020) present two calculation schemes for the variables based on specific periods of the year (all four interactive variables included). When the variables are calculated by climatological means, the variable always represents the same quarter or month. Thus, the variable always describes the same physiological and ecological phenomenon, but the definition of the variable is not valid anymore since, due to shifts within the annual cycle, the variable may not always represent the true timing of, for example, the wettest quarter. In contrast, the definition is valid when the variables are calculated year‐by‐year since the true wettest quarter is always chosen. However, since the wettest quarter may exhibit interannual variations, it may in 1 year represent precipitation in autumn and in the subsequent year precipitation in spring. Thus, the statistical link in SDM is not valid anymore since the BCV describes different ecological and physiological characteristics. In general, these findings are in accordance with the results of Bede‐Fazekas and Somodi (2020). It shows that their assumptions about the temporal context of future species distribution assessments are also valid for the calculation of BCVs within the recent period.

In the following, we will address these caveats to show that there are still factors to be considered when handling the interactive variables. In terms of the wettest quarter, the largest variability can be observed over the northeastern parts of Spain, the border area of Romania and Bulgaria, and the Caucasus region of Georgia, with an interannual shift of up to 4 months. This means that no clear rainy season can be defined for this region since it is shifted not only by months but also by seasons. For example, over the northeastern parts of Spain, there are no clear differences between BCV‐8 and BCV‐16 due to the calculation scheme, but regions with their maximum in spring and regions with their maximum in autumn are located next to each other. This can be attributed to the fact that the precipitation regime of the Spanish Mediterranean coast and its backcountry is mainly decoupled from the westerly wind drift, and precipitation events depend on weather conditions with an easterly or northerly wind component (Rodrigo & Trigo, 2007). The lack of a barrier along the Catalan coast allows these weather conditions to penetrate further inland (Cortesi et al., 2014). The relative frequency of these weather conditions is subject to an annual cycle with maxima in summer and minima in winter. Since the Azores High over the Iberian Peninsula has a stabilizing effect in summer, these weather conditions are rarely associated with precipitation in summer, and precipitation maxima are shifted to the transitional seasons. Especially in the backcountry, convective precipitation events provide precipitation maxima in spring since the thermal land‐sea contrast in the Mediterranean region is decreasing while the Azores High has not yet reached the stabilizing characteristics of the summer season (Esteban‐Parra et al., 1998). In contrast, subcontinental convective processes are negligible along the coast. Here, precipitation maxima occur in autumn due to the high temperatures of the Mediterranean Sea and the resulting enhanced cyclogenesis (Rodrigo & Trigo, 2007). Depending on the intensity of these weather conditions, the boundary between the two precipitation regions fluctuates, resulting in interannual shifts of the wettest quarter by more than 4 months. Nevertheless, the precipitation differences between spring and autumn peaks within this region are rather small, although different weather conditions are responsible for precipitation generation. Thus, the influence on the BCV is rather small, although the statistical link is not valid, since it describes two completely different life cycles or phenological phases (e.g., juvenile stage in spring and senior stage in autumn).

In contrast, the northern part of Portugal shows only a small difference between both approaches with respect to the wettest quarter (about 2 months). According to the CLIM approach, the wettest quarter is in late autumn (October–December), while the BIO scheme selects mainly the December–February period. In autumn, the temperatures of the Atlantic Ocean are still high, and due to evaporation, a large amount of precipitable water enters the atmosphere. As the Azores High tends to weaken in autumn, the northern part of Portugal falls under the influence of westerly wind drift, and the precipitation pattern is mainly determined by Atlantic cyclones. Atlantic cyclones and a huge amount of precipitable water combined with orographic effects can lead to intense precipitation events in this area (Santos et al., 2017). Overall, due to sporadic extreme precipitation events, the climatologically wettest quarter occurs in late autumn, while mid‐winter represents the quarter that quantitatively exhibits the highest precipitation amounts. Thus, although the statistical link is almost identical, huge differences of up to 260 mm can be observed between the CLIM and BIO calculation schemes.

The same can be observed for temperature in Russia, northeast of the Sea of Azov. Due to the high degree of continentality, a relatively small shift of 2 months leads to a difference between the BIO and CLIM approaches of up to 13°C.

Overall, 55.4% of the study area exhibits the same wettest quarter in both calculation schemes. For the driest quarter, the agreement is 68.2%, but with higher differences for absolute temperatures (BCV‐9; see Appendix S1.4). Here, the study area is divided into a northern and a southern part. In the northern part of the study area, BCV‐9 and precipitation of the driest quarter (BCV‐17) of the CLIM approach are notably smaller than under consideration of the BIO calculation scheme. In contrast, temperatures in the southern part are notably higher for CLIM, whereas differences with respect to precipitation hardly exist. An even greater agreement with respect to the selected quarter can be seen for the hottest (98.2%) and coldest quarter (99.7%). Therefore, differences in precipitation and temperature between both calculation schemes barely exist (Appendices S1.5 and S1.6).

We showed that there could be large differences between the two calculation schemes within the study area, confirming the analysis of Bede‐Fazekas and Somodi (2020). However, we also showed that the discontinuities resulting from the different calculation schemes affect the BCVs to a greater or lesser extent. In the northeastern parts of Spain, where large interannual shifts of the wettest season occur, the effect on the respective BCVs is rather small, whereas in the northern parts of Portugal (precipitation) and southeastern parts of Russia (temperature), relatively small shifts lead to large differences between the CLIM and BIO calculation schemes. This means that although the statistical link within the BIO approach is not valid and different physiological and ecological characteristics are described, the effect on BCV may be rather small, and conversely, small shifts in quarters may have a substantial influence on BCV, although the physiological and ecological phenomena are almost the same.

### 4.2 Differences between datasets

The ecological hypotheses tested by SDMs are affected by inconsistencies in climatic datasets, as they represent another source of uncertainty (Morales‐Barbero & Vega‐Álvarez, 2019). However, Watling et al. (2014) argue that neither model performance nor spatial predictions vary significantly due to different climate inputs and that the results of SDMs are more influenced by the modeling algorithm. As their study is limited to species in Florida, which is topographically plain, mountainous regions, which obviously show strong discrepancies between datasets (e.g., Cerasoli et al., 2022; Morales‐Barbero & Vega‐Álvarez, 2019), are not present in the analysis. Jimenez‐Valverde et al. (2021) also found higher inter‐model discrepancies for precipitation than for temperatures, with annual mean temperatures (BCV‐1) generally showing the highest agreement, which is also confirmed in the present study. Cerasoli et al. (2022) point out that BCVs representing variability (BCV‐2, BCV‐4, and BCV‐15) show larger differences between datasets than seasonal or annual mean values. Again, we found considerable differences in the seasonal means, but this is mainly due to the intra‐annual shifts of the season. Overall, inter‐model differences arise when the dataset is based on the interpolation methods of a meteorological station network and the respective region only exhibits a small number of stations. In this case, climate dataset based on quasi‐mechanistic downscaling or remote sensing are more accurate (Waltari et al., 2014). Since WorldClim provides a high‐quality dataset in station‐rich regions such as Europe (Fick & Hijmans, 2017), differences in our study area cannot be attributed to this issue. Furthermore, Jiménez‐Valverde et al. (2021) point out that differences between datasets diminish when the resolution is reduced. However, our studies are based on the same resolution as the “low resolution model” of Jiménez‐Valverde et al. (2021), and inter‐model differences are still present.

In the present study, all examined datasets represent the BCVs well. Since E‐OBS does not provide data for the entire study area, comparisons are made only for the E‐OBS grid boxes. For means, E‐OBS shows the greatest agreement with WorldClim for BCV‐1, but the tails are shifted to higher values with respect to extremes. In contrast, ERA5‐Land has the largest deviation from the mean, but the range of temperatures is similarly represented, while CRU has the largest absolute deviations from the WorldClim dataset. Overall, most deviations from all datasets are limited to ±2°C, and mean deviations are close to zero (without outliers). For BCV‐12, CRU has the highest agreement with respect to means and extremes, and ERA5‐Land shows notably higher precipitation amounts than WorldClim. Thus, the mean deviation of ERA5‐Land is 57 mm and covers the range from −218 to 357 mm, while the mean deviation of CRU is 2 mm, and the deviations range from −122 to 129 mm (without outliers). For BCV‐8, ERA5‐Land shows the highest general agreement with WorldClim for both means and extremes, and E‐OBS shows the lowest agreement, although the deviations have the smallest range (without outliers). Therefore, from a statistical point of view, no clear recommendation can be made, as all datasets have strengths and weaknesses.

Regarding spatial representation, the differences increase in complex terrain, especially for BCVs based on precipitation (amounts or periods). Differences between the GSORW‐Data and the reference of up to 2000 mm can be observed for BCV‐12 within the alpine region. Compared to WorldClim, all datasets also show notably lower annual precipitation amounts in the northern parts of Portugal and adjacent areas of Spain. As already seen within the statistical evaluation, ERA5‐Land exhibit comprehensive higher values of BCV‐12. With respect to the temperature of the wettest quarter (BCV‐8), all datasets show areas of high deviations west of the line between the Gulf of Lion and the German North Sea coast. This is due to the very heterogeneous characteristics of the rainy season within this area. Furthermore, large negative deviations can also be observed northeast of the Black Sea in ERA5‐Land and E‐OBS, while CRU generally agrees with WorldClim's BCV‐8. For annual mean temperatures (BCV‐1), heterogeneity is present for all datasets. ERA5‐Land, E‐OBS, and, to some extent, CRU show higher temperatures than WorldClim over much of Europe, while underestimating temperatures over the Maghreb and the Middle East. The locations of the highest deviations are spread mainly over the Alps and the eastern parts of the study area. Temperatures in the Alps are generally underestimated, with the largest deviations for CRU (−10°C) in the Italian Alps, whereas the highest deviations are in the southwestern part of Turkey (ERA5‐Land), east of the Black Sea (E‐OBS), or Armenia (CRU). All locations with the highest deviations (negative or positive) have in common that they prevail in mountainous regions.

From a technical point of view, all datasets have advantages and disadvantages. The BioClim dataset of WorldClim provides by far the highest spatial resolution, but BCVs are only available as means for the climatological period 1970–2000 and not as time series. For the same period, however, WorldClim offers six monthly averaged climatological variables and TMIN, TMAX, and PRE for the period 1960–2021 with a monthly temporal resolution. Thus, BCVs can be calculated for missing periods or on an annual temporal resolution. If SDM is performed using only BCVs, WorldClim provides a very suitable dataset. However, the recent trend within the SDM community is to train models using more (bio)climatological variables than just the BCVs. Variables beyond the set of 35 BCVs (e.g., growing degree days) can also be obtained from the CliMond organization (Kriticos et al., 2014). The website of the CliMond organization (www.climond.org) collects fully described and readily available predictors for SDM. Below, we list some studies that use variables beyond BCVs.

For example, Title and Bemmels (2018) include 16 climatic and two topographic variables (ENVIREM) to determine species distributions, as these variables are very likely to be directly related to ecological or physiological processes. The calculation of these variables requires data that is not available in WorldClim for all time periods. Kriticos et al. (2014) introduce five modified BCVs based on principal component analysis applied to the existing 35 BCVs. These five variables explain 90% of the variation in the 35 BCVs, but these variables should not be used in combination with the original BCVs. In addition, SDMs based on these five variables cannot be applied for climate change studies or for comparative analysis of datasets. Stewart et al. (2021) consider climate extremes to improve predicted distributions, particularly at plant range edges. Bailey and van de Pol (2016) expect changes in the frequency and magnitude of extremes to drive more drastic shifts in species distributions than changes in mean climate. As extreme weather events are short‐lived but are thought to have notable effects on plant distribution (Walter et al., 2013), monthly time series data are not sufficient. Furthermore, Booth (2022) suggests using seasonal or monthly variables based on the water balance as used by the BIOCLIM team in 1999 and acknowledges that several recent studies have also included evaporation measures. However, due to the daily scale of E‐OBS and CRU, both datasets are suitable when considering extremes for SDM, but when building more complex models that include the water balance system, ERA5(‐Land) or an equivalent reanalysis dataset should be the choice. When conducting studies based on BCVs and more complex variables, all variables should be taken from one dataset, as the processes mapped are then consistent from a climatological point of view.

## 5 CONCLUSIONS

The study presented here shows that the WorldClim dataset provides an adequate data basis when the BCVs are the only climatological variables to be used in SDM analysis. However, if additional climatological variables are to be considered in SDM studies, other datasets should be considered for the sake of data consistency. All evaluated datasets provide high‐quality data, and thus the choice of dataset for SDM depends on other characteristics, such as data availability for the region of interest, temporal and spatial resolution, or availability of climatological variables critical for species distribution. Here, ERA5‐Land is a good choice, as this dataset contains by far the most climatological data for the global domain, as well as a high temporal and spatial resolution.

Since the datasets examined here do not provide BCVs, these must first be calculated based on the available data. As the definition of BCVs leaves some room for maneuver, especially with regard to temporal reference periods, researchers should consider beforehand exactly what goal they are pursuing when modeling species distributions or ecological niches. In terms of calculation schemes, differences occur, especially when BCVs represent precipitation, when the period of interest is determined by precipitation, or in complex terrain. However, no general recommendation can be made here, as we have found areas where large temporal shifts in the period of interest, such as the wettest quarter, have little effect on the variable and vice versa. In addition, we also found regions where the spatial pattern of the periods of interest resembles a small‐scale mosaic. This can also result in large spatial differences, especially with respect to the mean temperatures of, for example, the wettest quarter (BCV‐8), when areas with precipitation maxima in summer and winter are adjacent to each other. Thus, we comply with the recommendation of Booth (2022) that BCVs should be carefully evaluated with respect to temporal or spatial discontinuities within the respective study area and, if necessary, to remove variables with substantial differences. Another option would be to define fixed specific quarters based on phenological characteristics rather than climatological ones. However, this requires an understanding of the phenological characteristics of the species and its dependence on specific climatological conditions at specific times of the year. For example, if an annual plant requires precipitation within the juvenile stage, precipitation in spring (e.g., March–April–May) should be the variable of interest. Fixed quarters based on phenological characteristics ensure that both the statistical and the phenological link are valid. A similar assumption is provided by Booth (2022), who recommends fixed winter and summer periods as one option to overcome these discontinuities.

The question of which calculation scheme to use depends primarily on the scientific question, the species, and what is known about the natural limits of that species. One suggestion is that, if the natural limits of the species are known, absolute rather than mean maximum (BCV‐5) or minimum (BCV‐6) temperatures and year‐by‐year (BIO) calculation of BCVs may be better for immobile species (i.e., plants) as they better represent the biological limits of the species. In contrast, for mobile species (i.e., animals), climatological means (CLIM) should be considered since the distribution of the species is not limited by single events but by long‐term disturbances. In addition, we recommend to rely on the climatological calculation scheme if the goal of the study is to assess the future distribution or ecological niches of a species. Although this blurs the definition of the variables, since the actual wettest period of the year is not always selected, the statistical link is preserved. In our opinion, this is a prerequisite when models are transferred to future periods where intra‐annual relocations of, for example, the wettest quarter are more than likely. This is also in agreement with Bede‐Fazekas and Somodi (2020), who recommend using the static approach for BCV calculation to achieve better congruence between recent and future periods. In general, whatever approach is used to calculate BCVs, a detailed description of the calculation scheme (Bede‐Fazekas & Somodi, 2020) and a justification of why this scheme is used should be provided in each study.

In summary, BCVs are considered blessings in part as they have provided effective results for many hundreds of SDM analyses (Bradie & Leung, 2017). However, this blessing can turn into a curse, as the vague definition of the variables allows some leeway. Without deeper insight into the underlying calculation scheme, the use of BCVs implies that results from different studies are easily comparable, which is obviously not the case when different calculation schemes are used. Using the same set of BCVs and the same statistical model, different calculation schemes for SDM of the same species may produce different results, so comparability of results is limited at best.

## AUTHOR CONTRIBUTIONS

**Christian Merkenschlager:** Conceptualization (lead); data curation (lead); formal analysis (lead); investigation (lead); methodology (lead); validation (lead); visualization (lead); writing – original draft (lead); writing – review and editing (lead). **Freddy Bangelesa:** Formal analysis (supporting); methodology (supporting); validation (supporting); visualization (supporting); writing – original draft (supporting); writing – review and editing (supporting). **Heiko Paeth:** Funding acquisition (lead); project administration (lead); supervision (lead); writing – original draft (supporting); writing – review and editing (supporting). **Elke Hertig:** Conceptualization (supporting); funding acquisition (lead); investigation (supporting); methodology (supporting); project administration (lead); supervision (lead); writing – original draft (supporting); writing – review and editing (supporting).

## Supporting information


Appendix S1.
Appendix S2.

## Data Availability

The data that support the findings of this study are openly available in: WorldClim: https://www.worldclim.org/, Hijmans et al. (2005), Fick and Hijmans (2017), ERA5‐Land: https://cds.climate.copernicus.eu/, Muñoz Sabater (2019), E‐OBS: https://www.ecad.eu/; Cornes et al. (2018), CRU: https://crudata.uea.ac.uk/, Harris et al. (2020). Data openly available in a public repository that issues datasets with DOIs.
