# Supplementary material for: Blessing and curse of bioclimatic variables: A comparison of different calculation schemes and datasets for species distribution modeling within the extended Mediterranean area
Source: Ecol Evol. 2023 Sep 28;13(10):e10553. doi: 10.1002/ece3.10553 (PMC10534195; doi:10.1002/ece3.10553)
Supplement: Supplementary file 1 — Appendix S1. Appendix S2. [file ECE3-13-e10553-s001.docx]

**Supplementary**

**S1: Differences of BioClim variables due to calculation scheme**

**
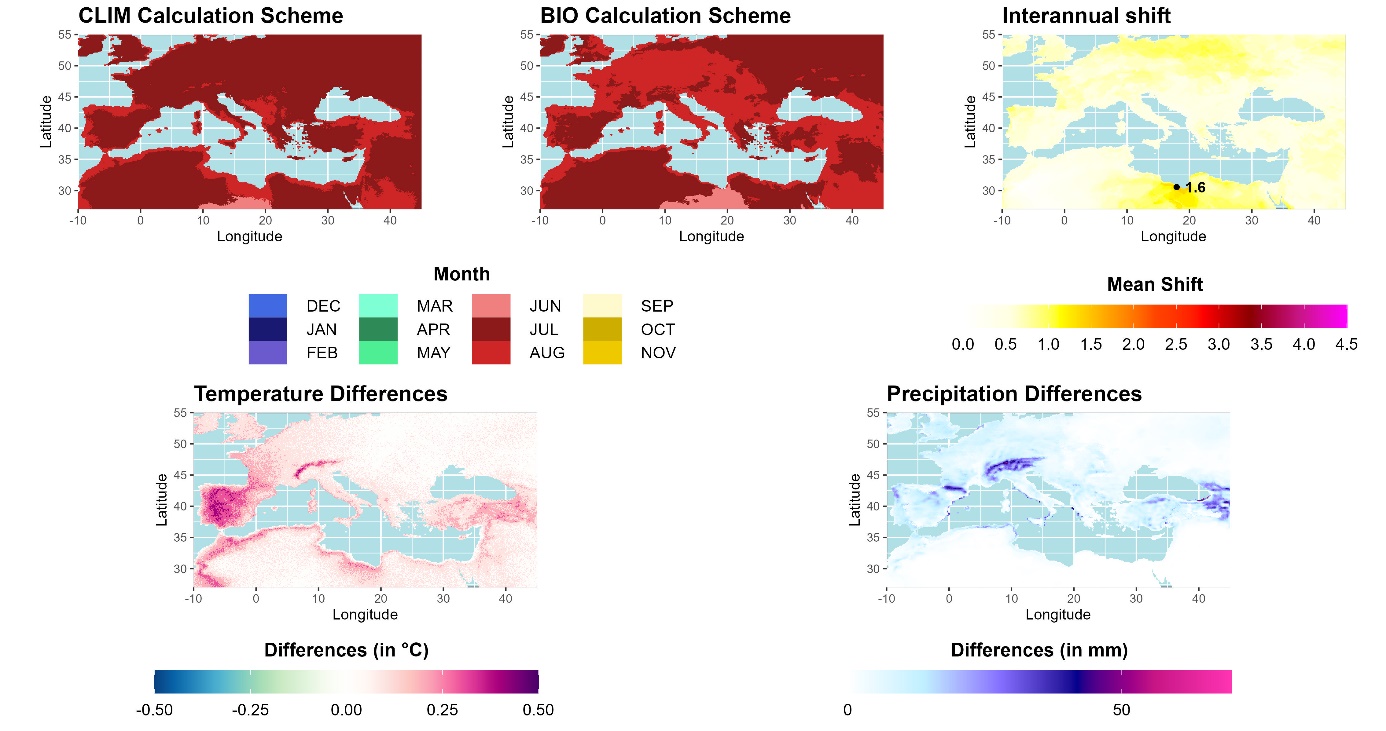
**

*S1.1:* ***BCV-5*** *Maximum Temperature of the warmest month. The upper figures represent the climatological months of the warmest month (CLIM, top left) and the month most frequently represents the warmest month due to the biological approach (BIO, top mid) of the ERA5-Land dataset (period 1970-2020). The figure at top right represents the mean interannual shift of the warmest month for the BIO calculation scheme (Maximum shift: black dot and number). Figures at the bottom show the differences between both approaches (BIO-CLIM) with respect to temperature (left) and precipitation (right).*

*
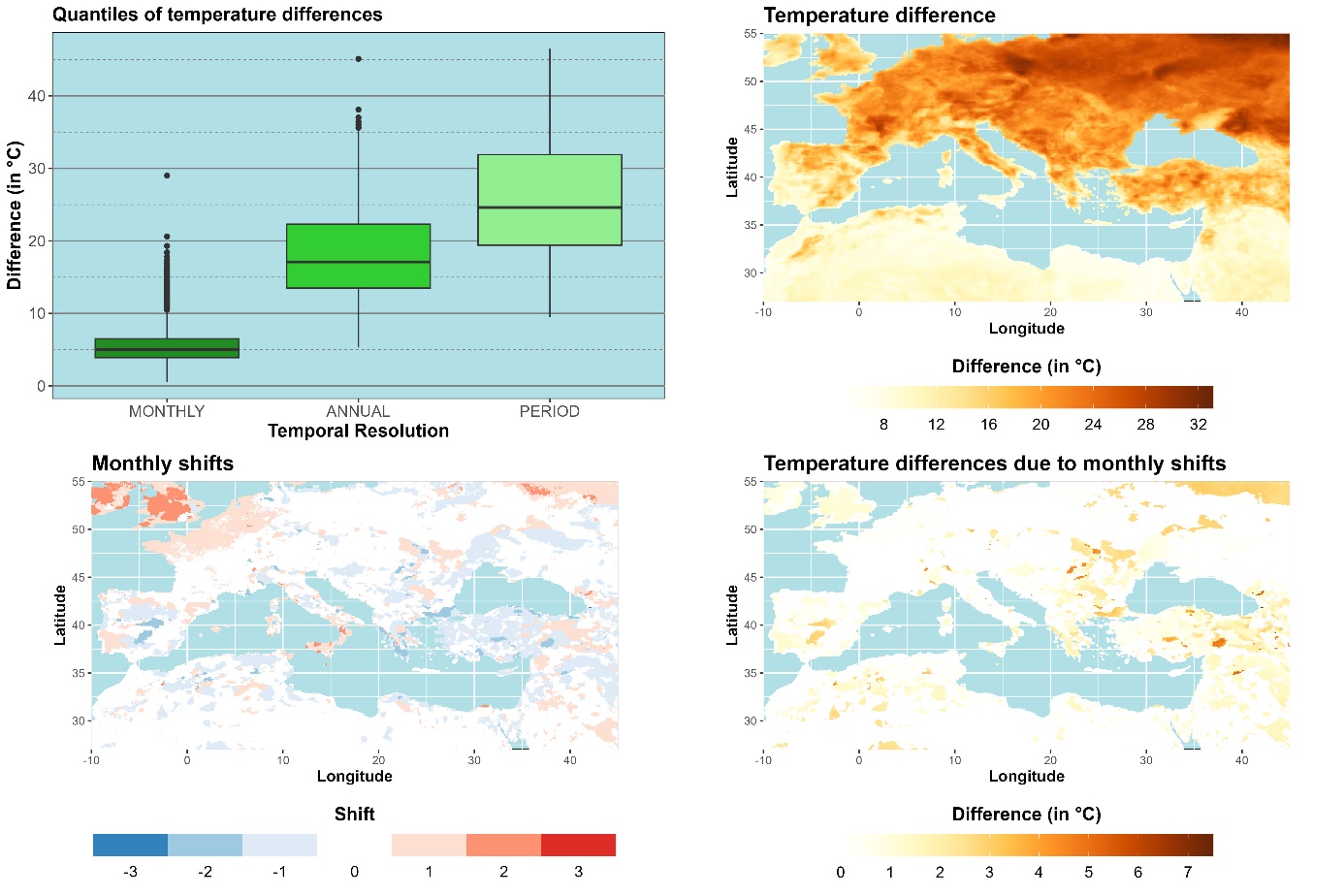
*

*S1.2:* ***BCV-6*** *Minimum temperatures of the coldest month: Differences between absolute and mean minimum temperatures (period 1970-2020) of ERA5-Land for different temporal scales (top, left) and regions (top right). The temporal shift of absolute and mean minimum temperature is presented at the bottom left. The shift-induced temperature difference of the mean minimum temperature is given at the bottom right.*

*
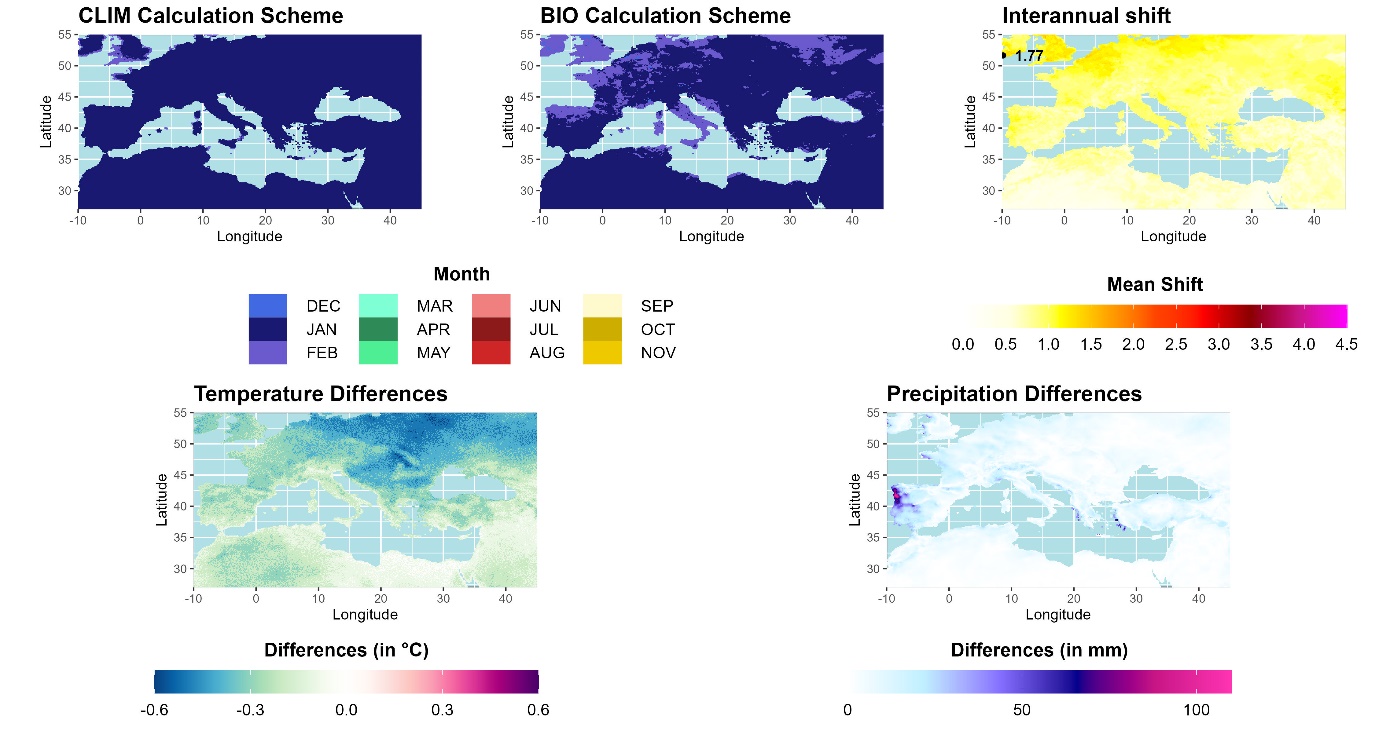
*

*S1.3:* ***BCV-6*** *Minimum Temperature of the coldest month. The upper figures represent the climatological months of the coldest month (CLIM, top left) and the month most frequently represents the coldest month due to the biological approach (BIO, top mid) of the ERA5-Land dataset (period 1970-2020). The figure at top right represents the mean interannual shift of the coldest month for the BIO calculation scheme (Maximum shift: black dot and number). Figures at the bottom show the differences between both approaches (BIO-CLIM) with respect to temperature (left) and precipitation (right).*

*
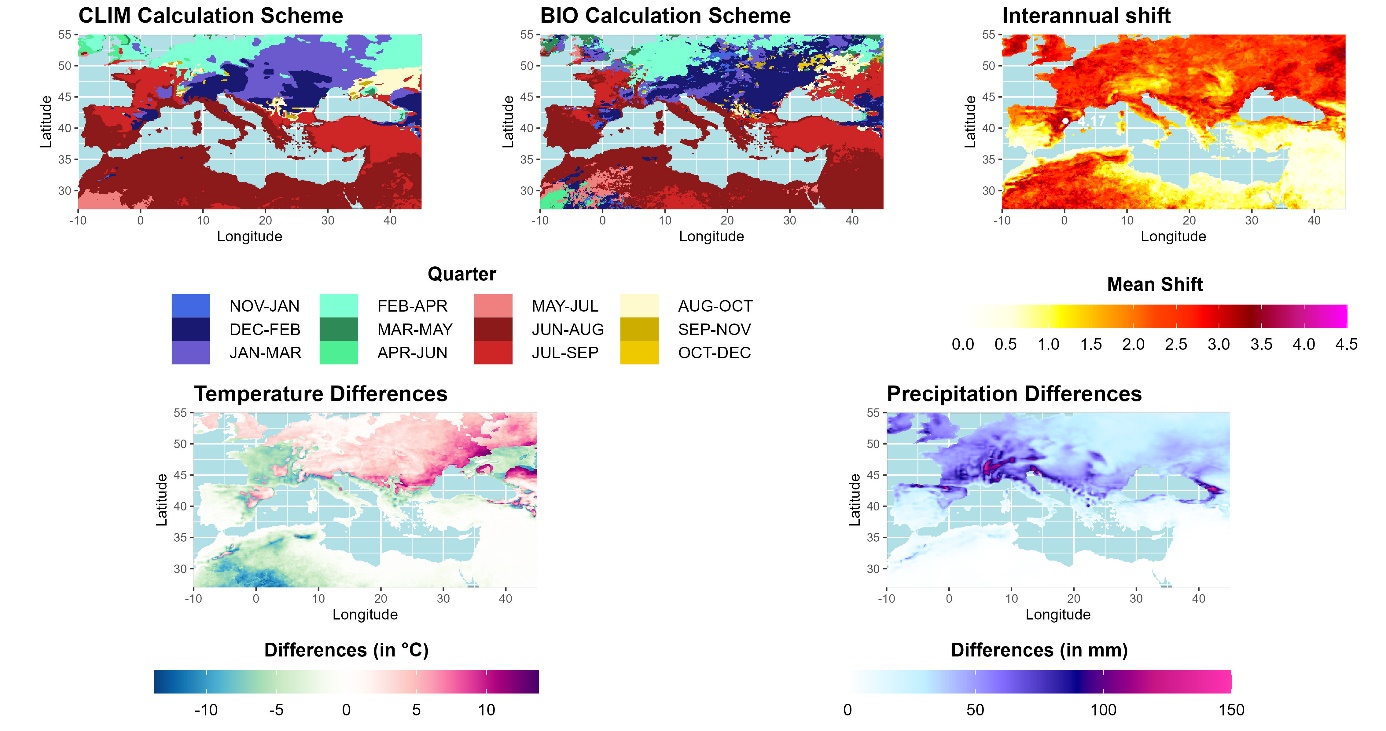
*

*S1.4:* ***BCV-9*** *and* ***BCV-17*** *Temperature and precipitation of the driest quarter: The upper figures represent the climatological quarters of the driest quarter (CLIM, top left) and the quarter most frequently represents the driest quarter due to the biological approach (BIO, top mid) of the ERA5-Land dataset (period 1970-2020). The figure at top right represents the mean interannual shift of the driest quarter for the BIO calculation scheme (Maximum shift: white dot and number). Figures at the bottom show the differences between both approaches (BIO-CLIM) with respect to temperature (left) and precipitation (right).*

*
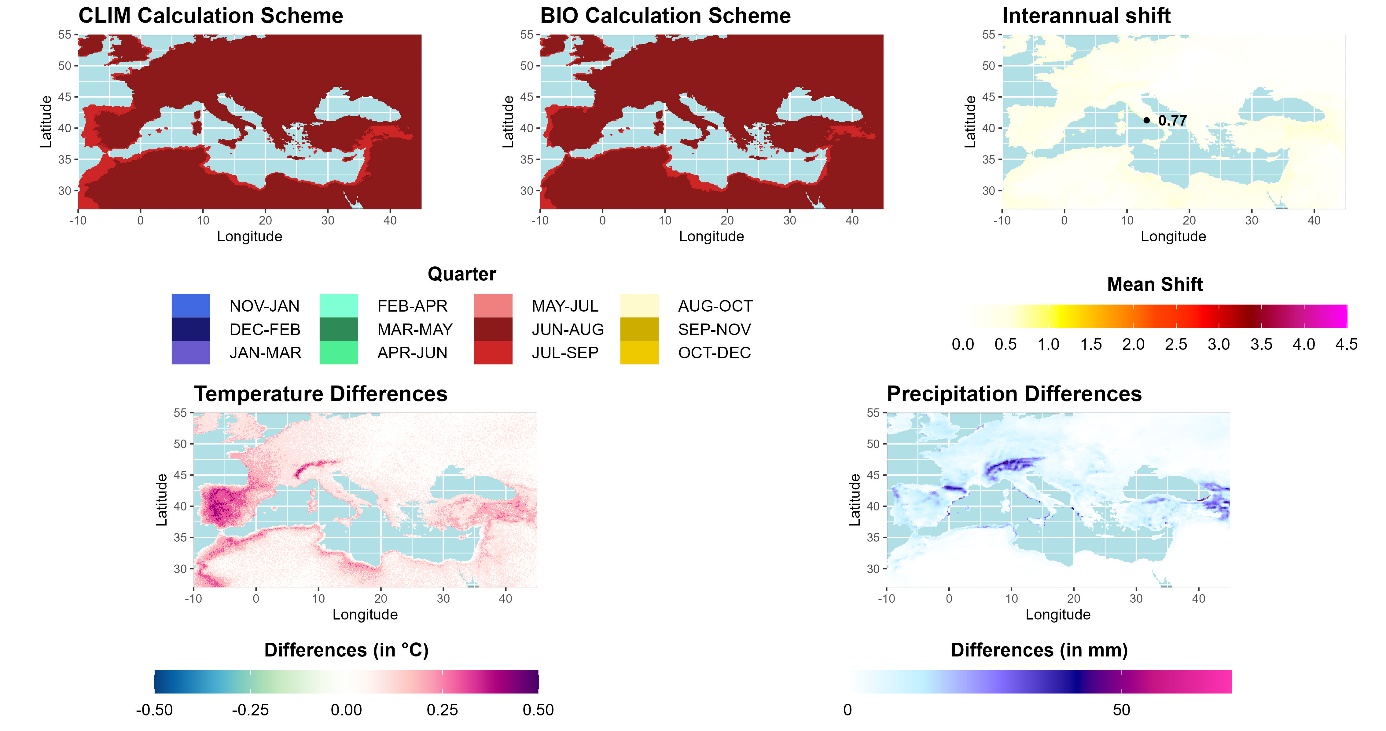
*

*S1.5:* ***BCV-10*** *and* ***BCV-18*** *Temperature and precipitation of the warmest quarter: The upper figures represent the climatological quarters of the warmest quarter (CLIM, top left) and the quarter most frequently represents the warmest quarter due to the biological approach (BIO, top mid) of the ERA5-Land dataset (period 1970-2020). The figure at top right represents the mean interannual shift of the warmest quarter for the BIO calculation scheme (Maximum shift: black dot and number). Figures at the bottom show the differences between both approaches (BIO-CLIM) with respect to temperature (left) and precipitation (right).*

*
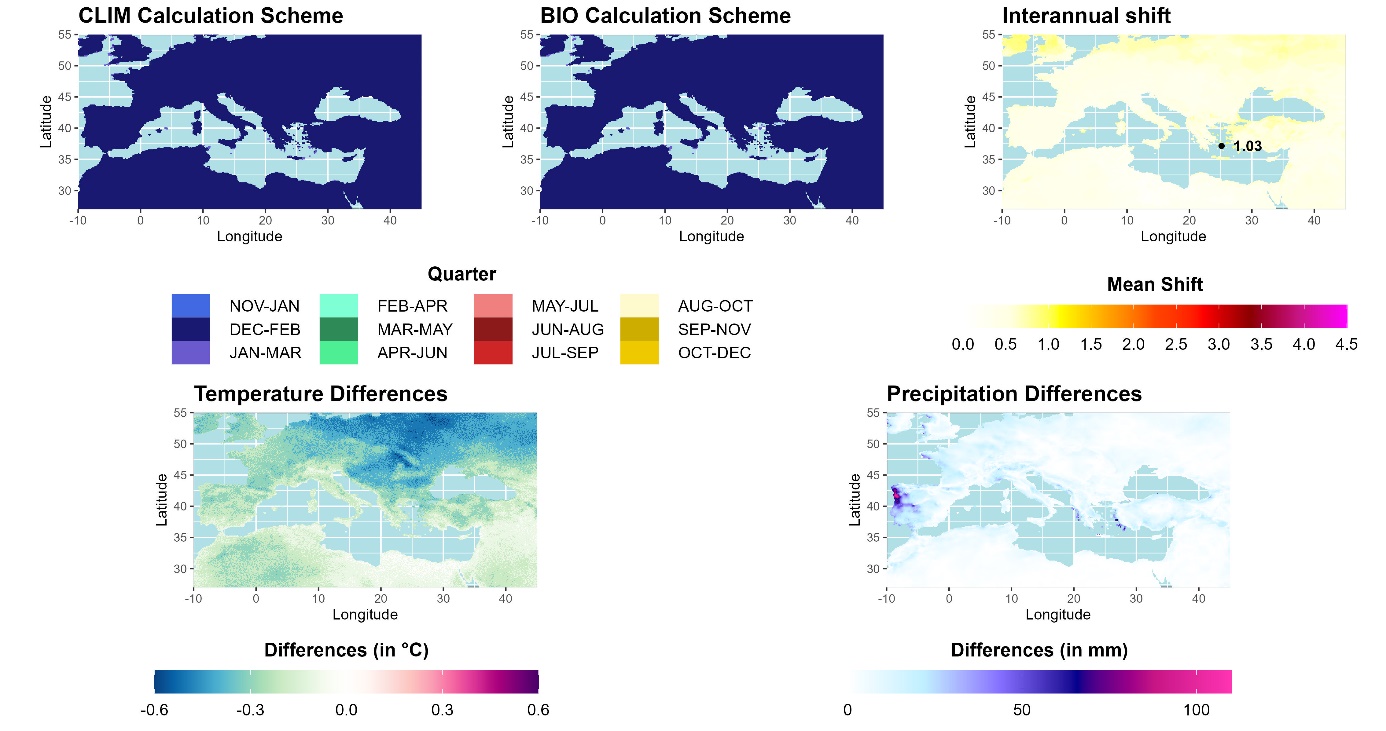
*

*S1.6:* ***BCV-11*** *and* ***BCV-19*** *Temperature and precipitation of the coldest quarter: The upper figures represent the climatological quarters of the coldest quarter (CLIM, top left) and the quarter most frequently represents the coldest quarter due to the biological approach (BIO, top mid) of the ERA5-Land dataset (period 1970-2020). The figure at top right represents the mean interannual shift of the coldest quarter for the BIO calculation scheme (Maximum shift: black dot and number). Figures at the bottom show the differences between both approaches (BIO-CLIM) with respect to temperature (left) and precipitation (right).*

*
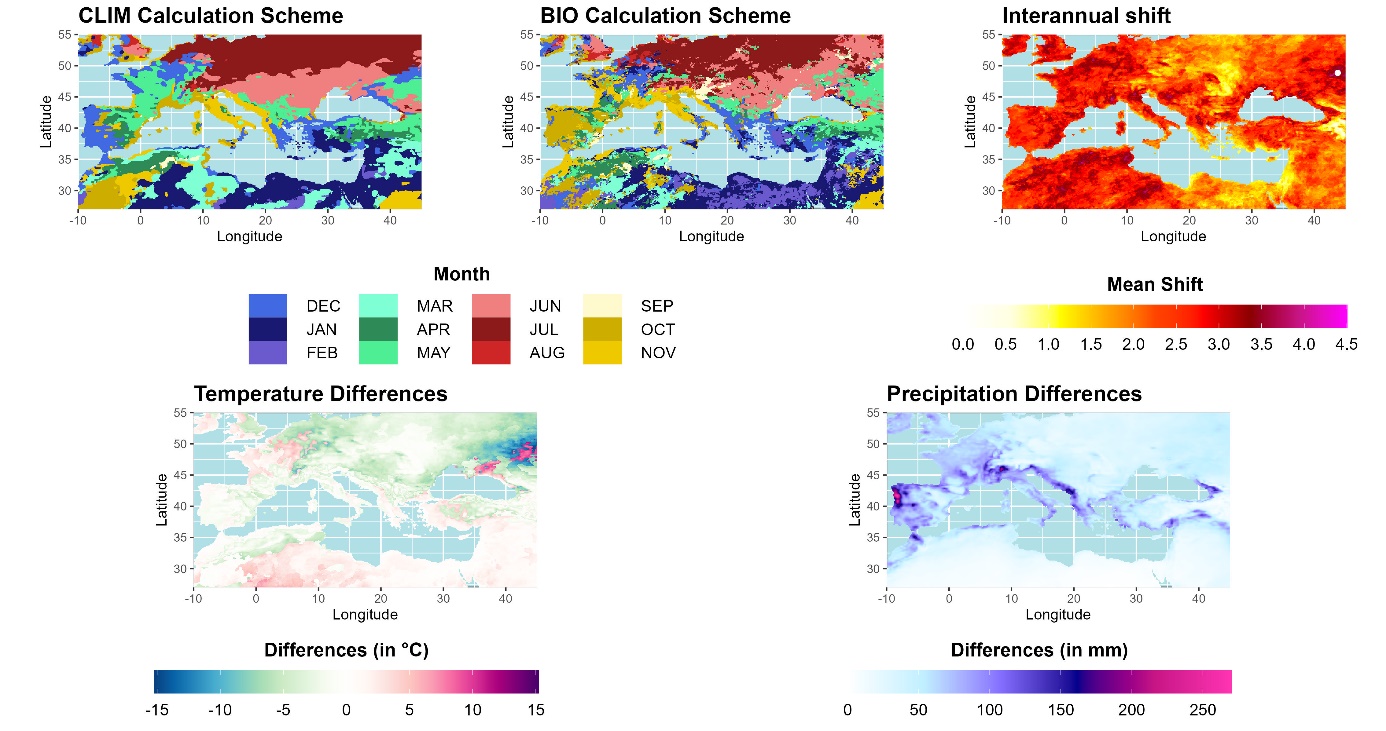
*

*S1.7:* ***BCV-13*** *Precipitation of the wettest month: The upper figures represent the climatological months of the wettest month (CLIM, top left) and the month most frequently represents the wettest month due to the biological approach (BIO, top mid) of the ERA5-Land dataset (period 1970-2020). The figure at top right represents the mean interannual shift of the wettest month for the BIO calculation scheme (Maximum shift: white dot and number). Figures at the bottom show the differences between both approaches (BIO-CLIM) with respect to temperature (left) and precipitation (right).*

*
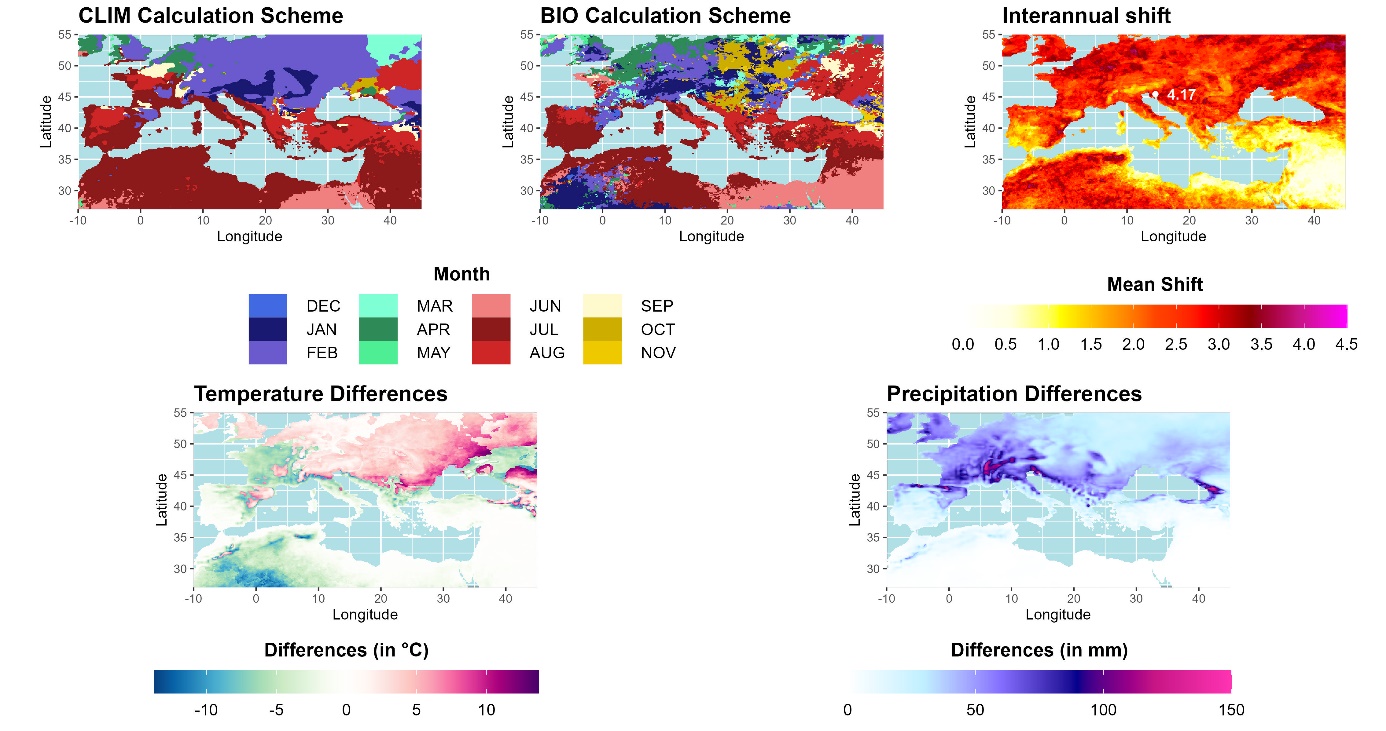
*

*S1.8:* ***BCV-14*** *Precipitation of the driest month: The upper figures represent the climatological months of the driest month (CLIM, top left) and the month most frequently represents the driest month due to the biological approach (BIO, top mid) of the ERA5-Land dataset (period 1970-2020). The figure at top right represents the mean interannual shift of the driest month for the BIO calculation scheme (Maximum shift: white dot and number). Figures at the bottom show the differences between both approaches (BIO-CLIM) with respect to temperature (left) and precipitation (right).*

**S2: Differences of BioClim variables due to datasets**

**
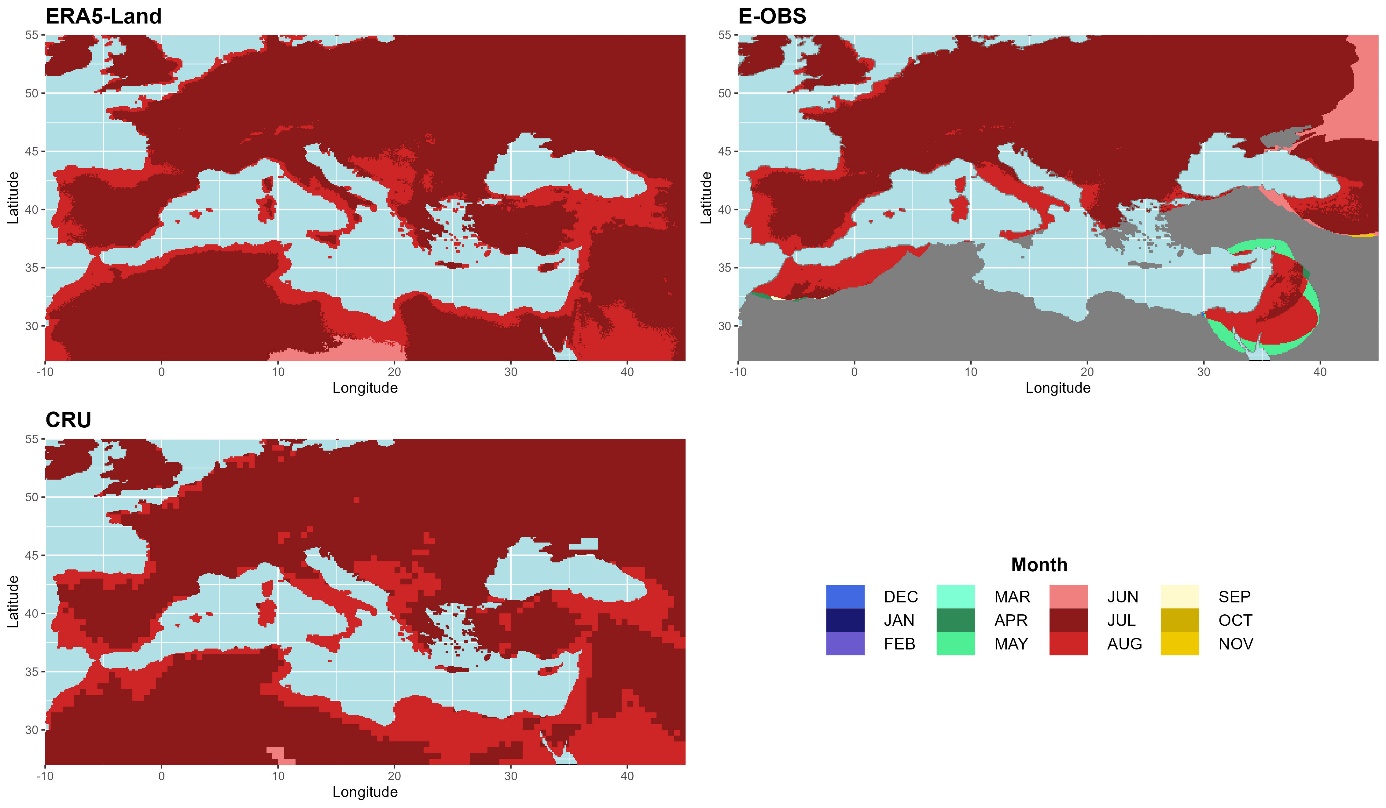
**

*S2.1:* ***BCV-5*** *Maximum temperature of the warmest month. The warmest month for ERA5-Land (top left), E-OBS (top right) and CRU (bottom left) for the period 1970-2020 (gray areas: No data).*

*
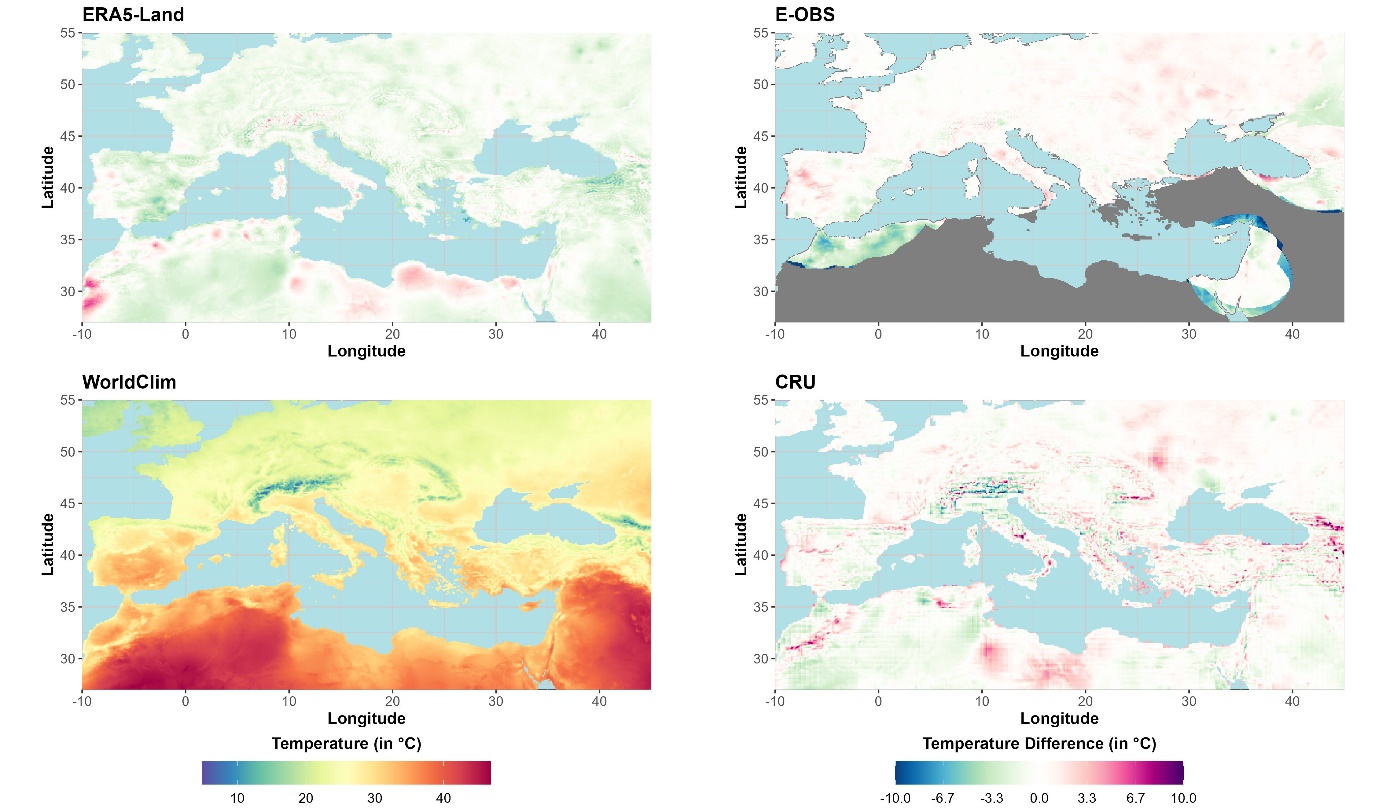
*

*S2.2:* ***BCV-5*** *Maximum temperature of the warmest month. The figure shows the temperatures of the WorldClim dataset (top left) and the temperature deviations (dataset - WorldClim) of ERA5-Land (bottom left), E-OBS (top right) and CRU (bottom right) for the period 1970-2000 (gray areas: No data).*

**
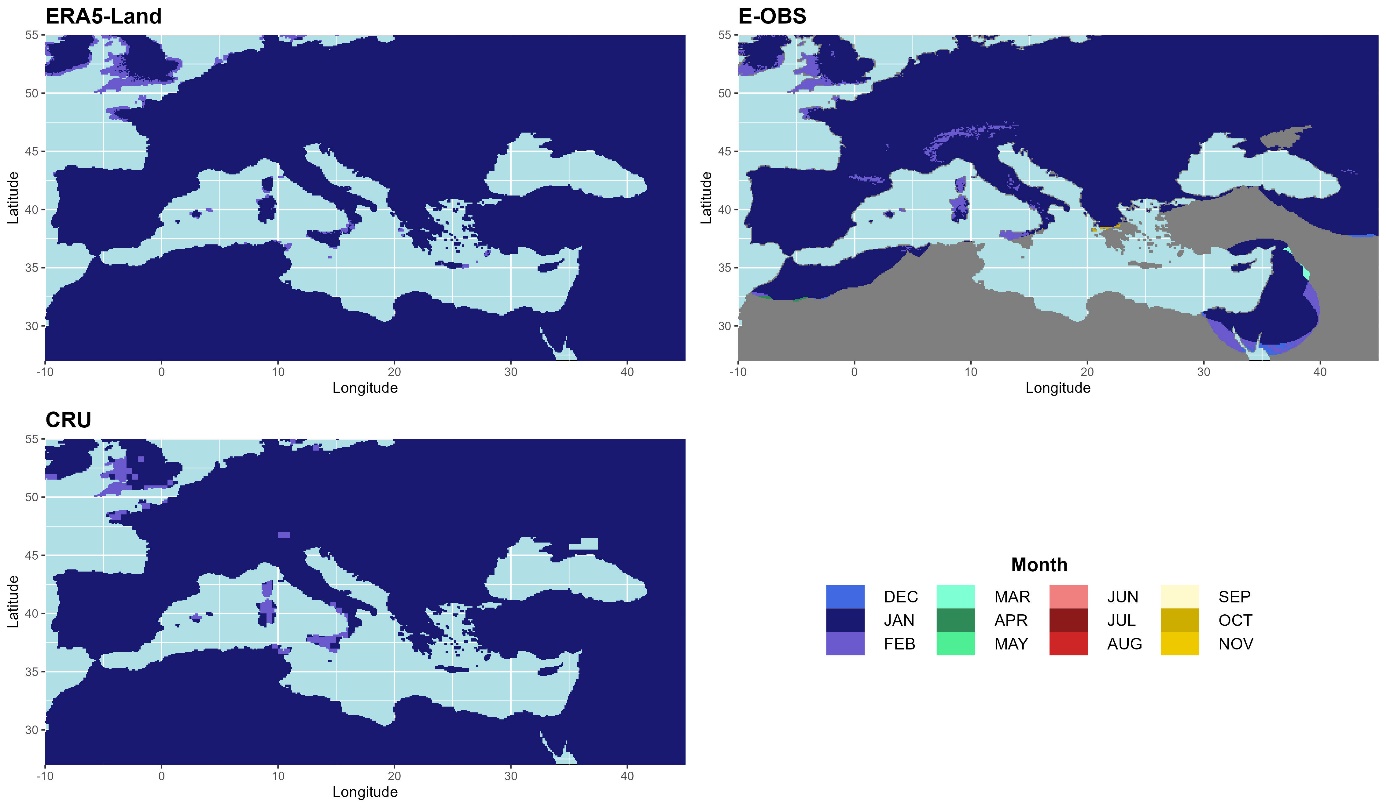
**

*S2.3:* ***BCV-6*** *Minimum temperature of the coldest month. The coldest month for ERA5-Land (top left), E-OBS (top right) and CRU (bottom left) for the period 1970-2020 (gray areas: No data).*

*
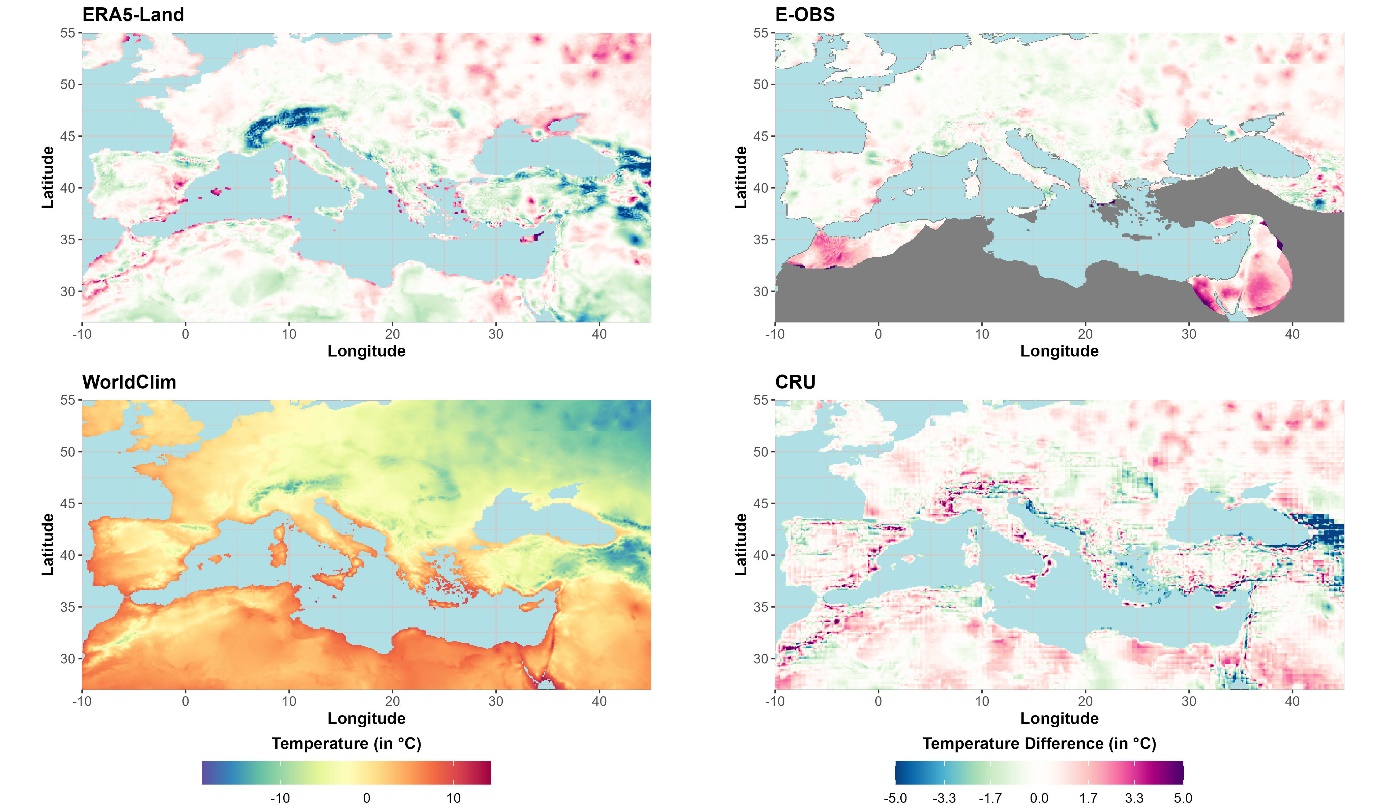
*

*S2.4:* ***BCV-6*** *Minimum temperature of the coldest month. The figure shows the temperatures of the WorldClim dataset (top left) and the temperature deviations (dataset - WorldClim) of ERA5-Land (bottom left), E-OBS (top right) and CRU (bottom right) for the period 1970-2000 (gray areas: No data).*

*
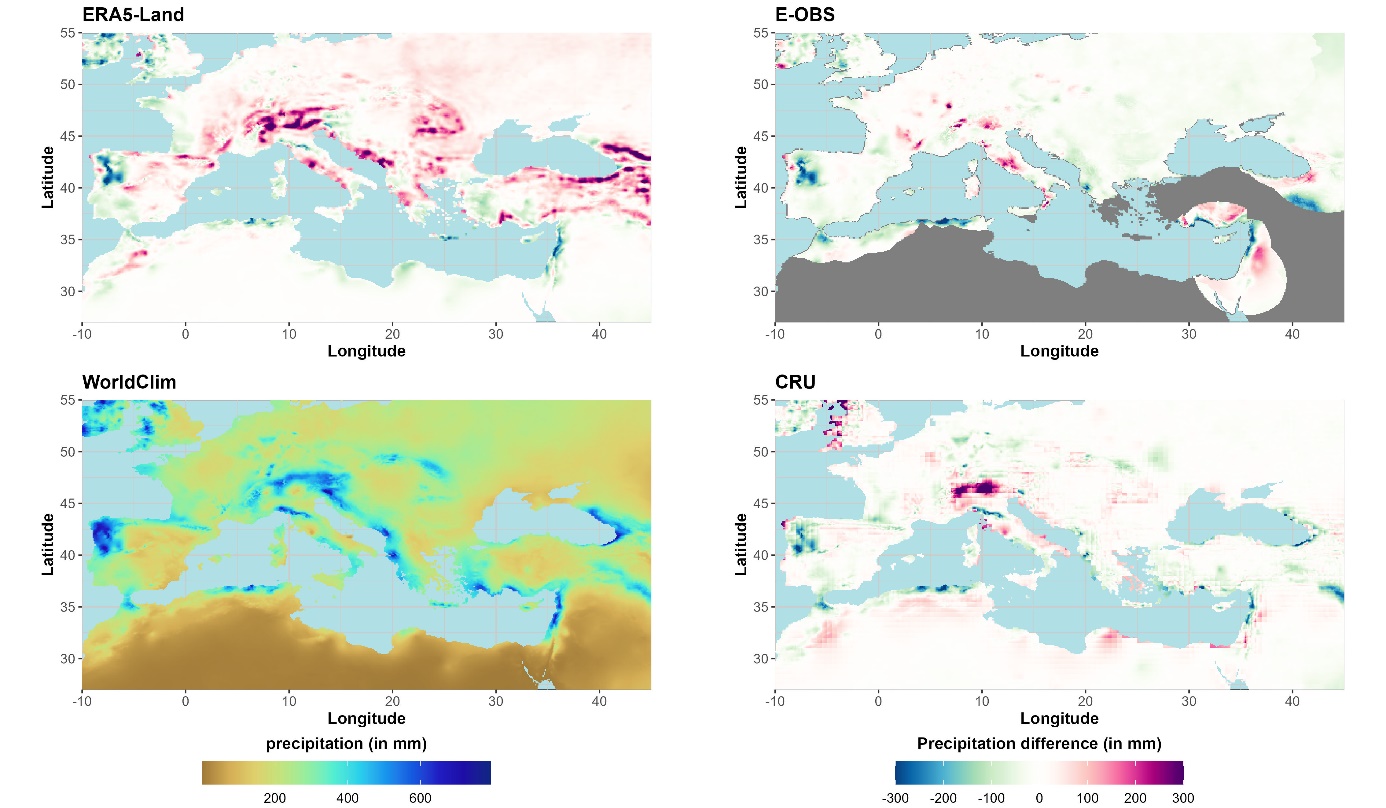
*

*S2.5:* ***BCV-16*** *Precipitation of the wettest quarter. The figure shows the precipitation amounts of the WorldClim dataset (top left) and the precipitation deviations (dataset - WorldClim) of ERA5-Land (bottom left), E-OBS (top right) and CRU (bottom right) for the period 1970-2000 (gray areas: No data).*

*
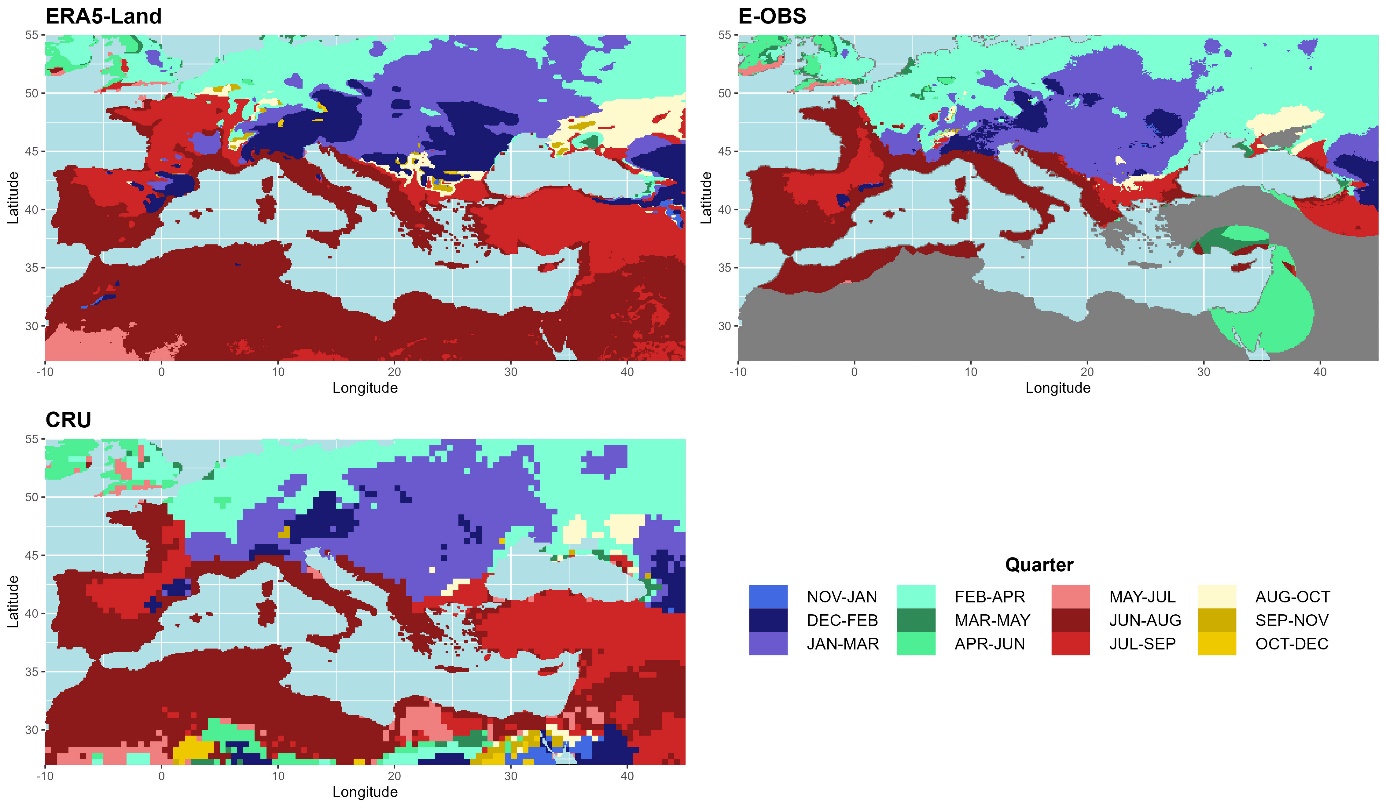
*

*S2.6:* ***BCV-9*** *and* ***BCV-17*** *Temperature and precipitation of the driest quarter. The driest quarter for ERA5-Land (top left), E-OBS (top right) and CRU (bottom left) for the period 1970-2020 (gray areas: No data).*

*
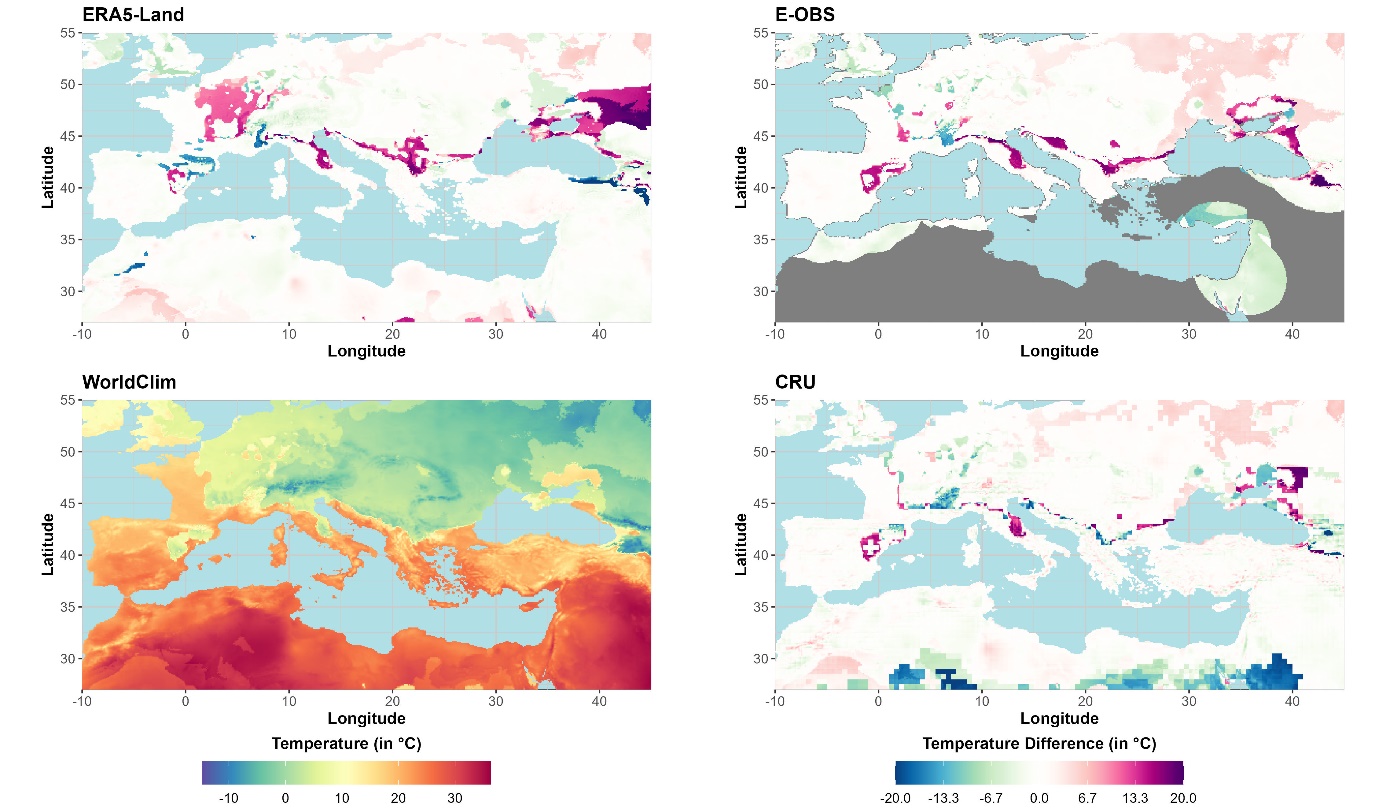
*

*S2.7:* ***BCV-9*** *Temperature of the driest quarter. The figure shows the temperatures of the WorldClim dataset (top left) and the temperature deviations (dataset - WorldClim) of ERA5-Land (bottom left), E-OBS (top right) and CRU (bottom right) for the period 1970-2000 (gray areas: No data).*

*
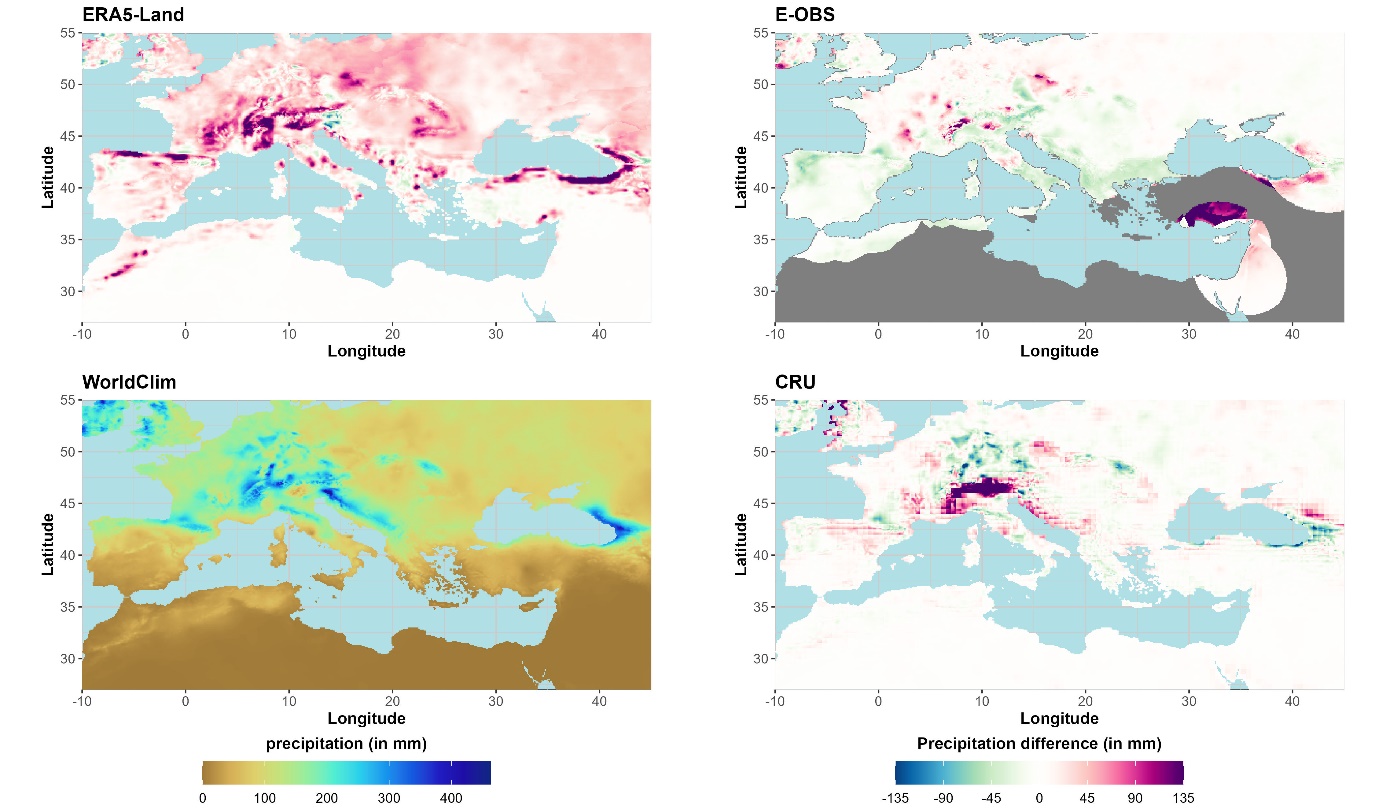
*

*S2.8:* ***BCV-17*** *Precipitation of the driest quarter. The figure shows the precipitation amounts of the WorldClim dataset (top left) and the precipitation deviations (dataset - WorldClim) of ERA5-Land (bottom left), E-OBS (top right) and CRU (bottom right) for the period 1970-2000 (gray areas: No data).*

*
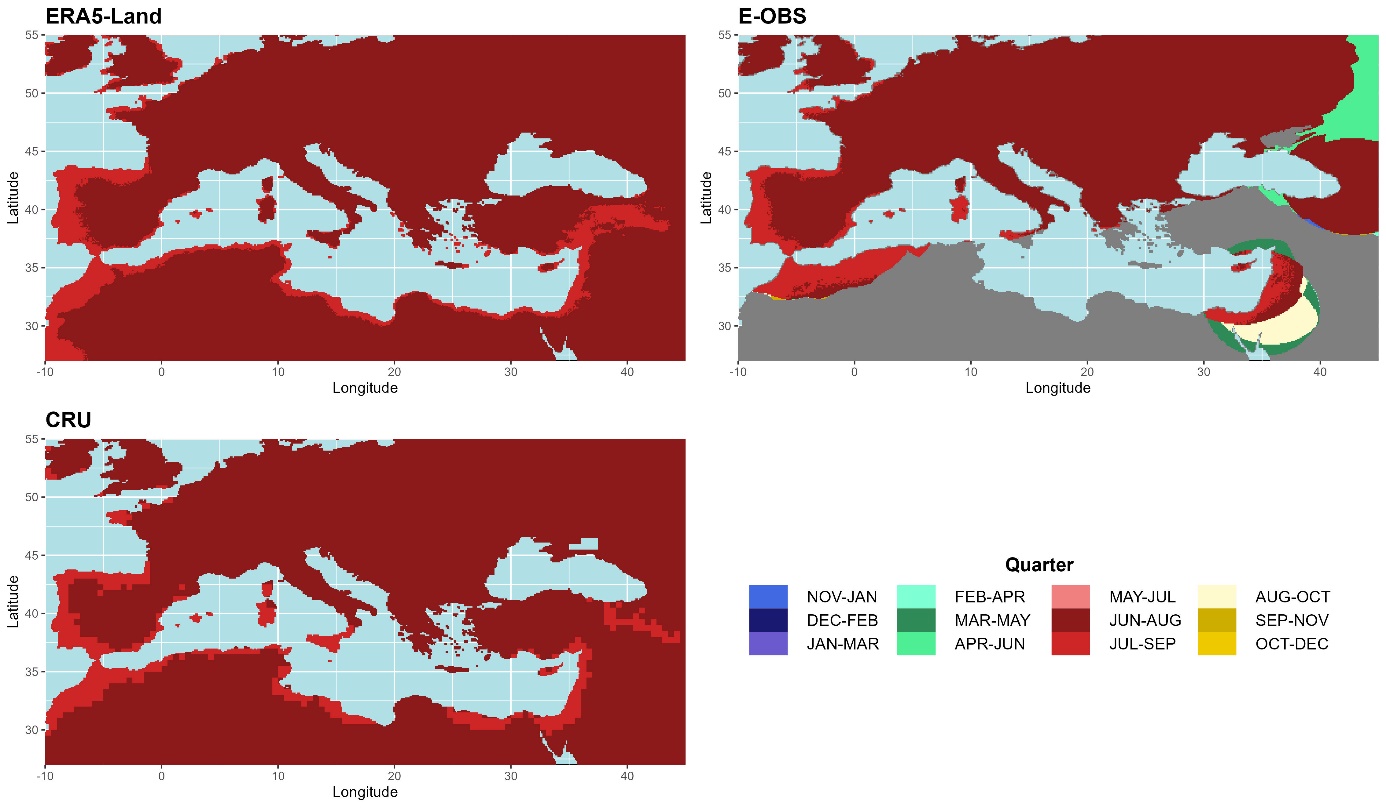
*

*S2.9:* ***BCV-10*** *and* ***BCV-18*** *Temperature and precipitation of the warmest quarter. The warmest quarter for ERA5-Land (top left), E-OBS (top right) and CRU (bottom left) for the period 1970-2020 (gray areas: No data).*

*
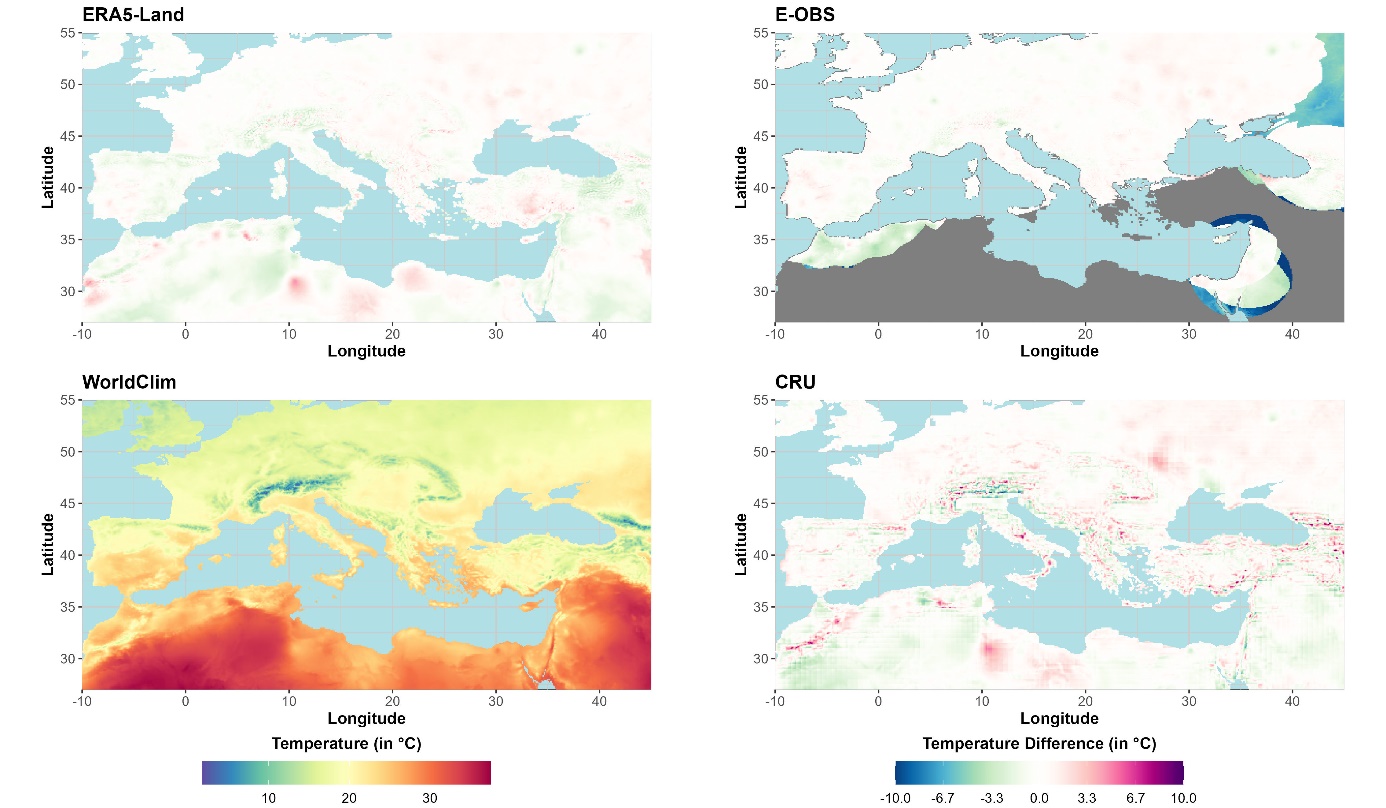
*

*S2.10:* ***BCV-10*** *Temperature of the warmest quarter. The figure shows the temperatures of the WorldClim dataset (top left) and the temperature deviations (dataset - WorldClim) of ERA5-Land (bottom left), E-OBS (top right) and CRU (bottom right) for the period 1970-2000 (gray areas: No data).*

*
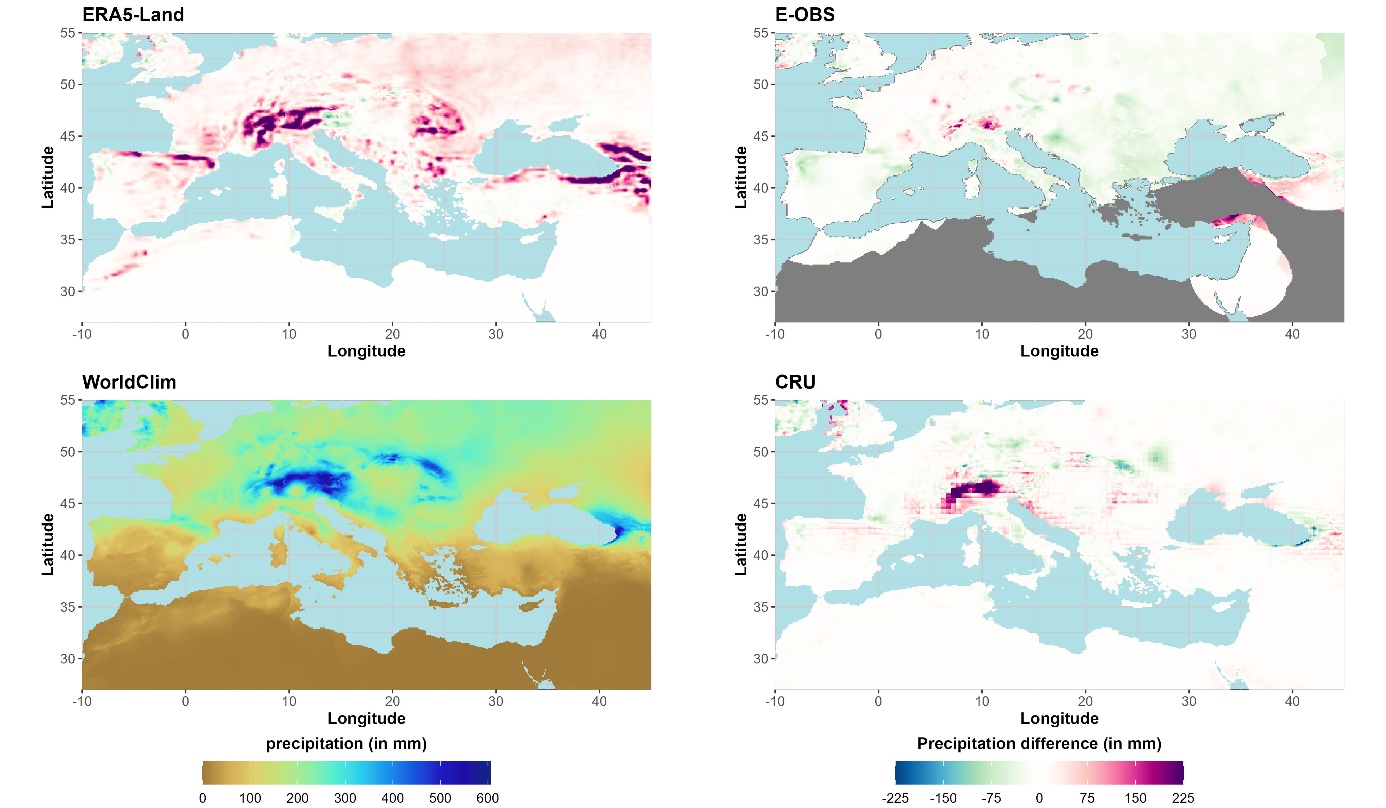
*

*S2.11:* ***BCV-18*** *Precipitation of the warmest quarter. The figure shows the precipitation amounts of the WorldClim dataset (top left) and the precipitation deviations (dataset - WorldClim) of ERA5-Land (bottom left), E-OBS (top right) and CRU (bottom right) for the period 1970-2000 (gray areas: No data).*

*
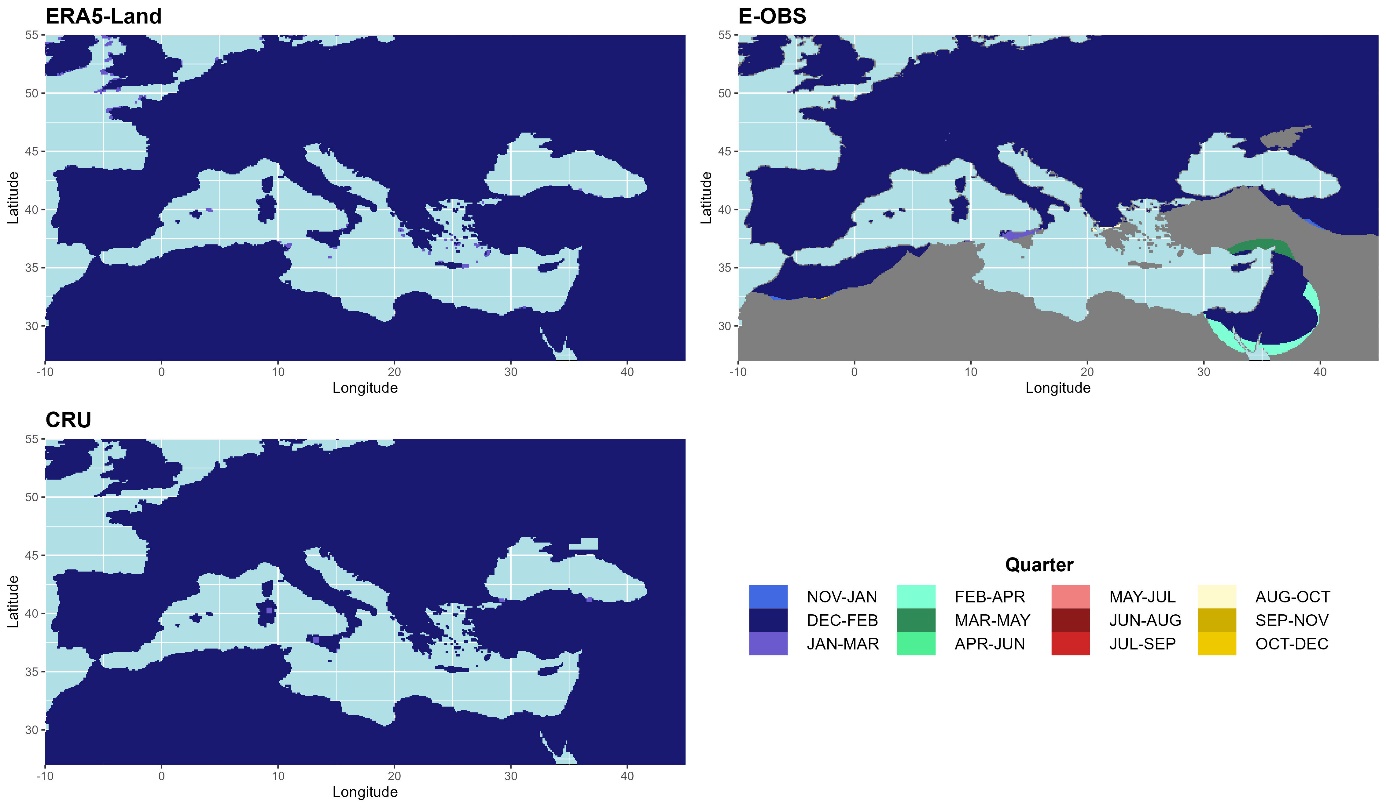
*

*S2.12:* ***BCV-11*** *and* ***BCV-19*** *Temperature and precipitation of the coldest quarter. The coldest quarter for ERA5-Land (top left), E-OBS (top right) and CRU (bottom left) for the period 1970-2020 (gray areas: No data).*

*
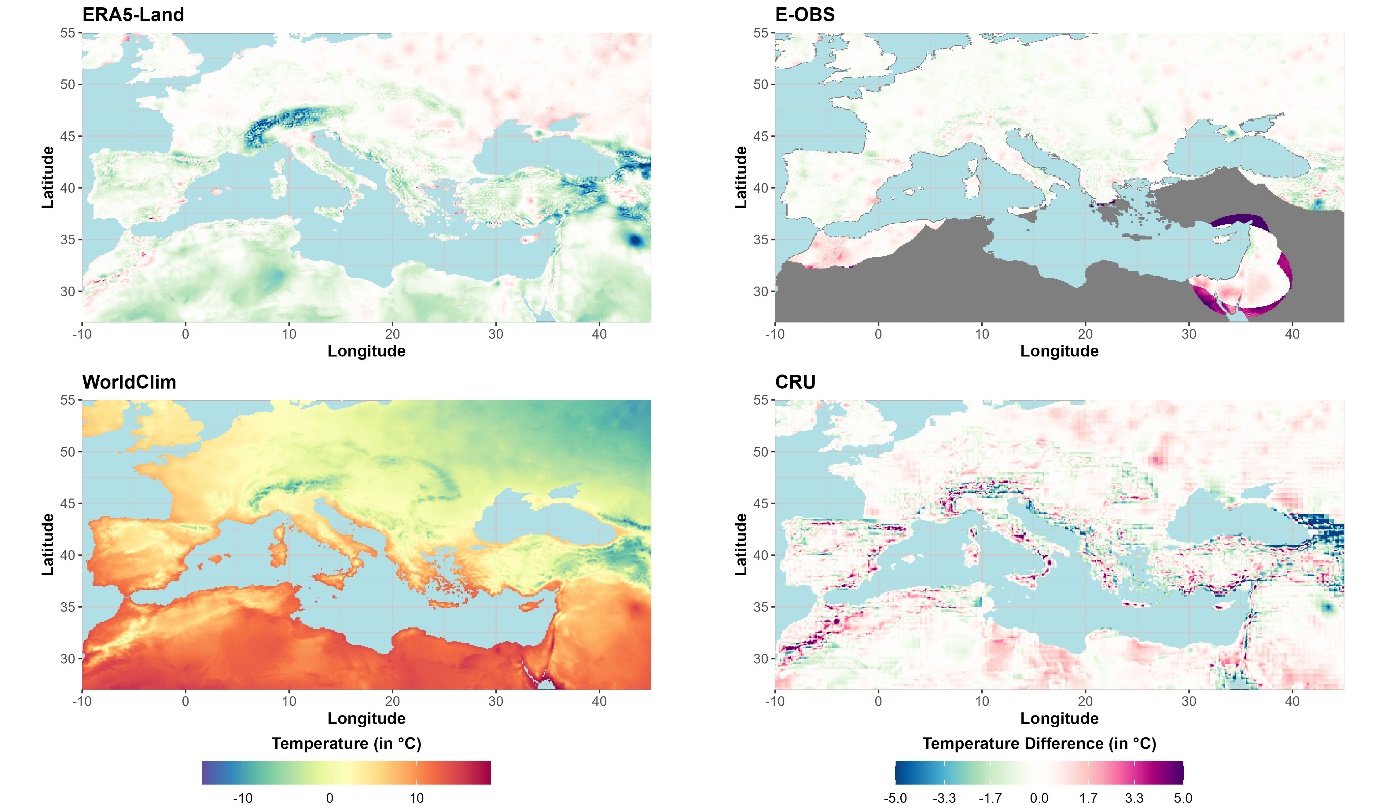
*

*S2.13:* ***BCV-11*** *Temperature of the coldest quarter. The figure shows the temperatures of the WorldClim dataset (top left) and the temperature deviations (dataset - WorldClim) of ERA5-Land (bottom left), E-OBS (top right) and CRU (bottom right) for the period 1970-2000 (gray areas: No data).*

*
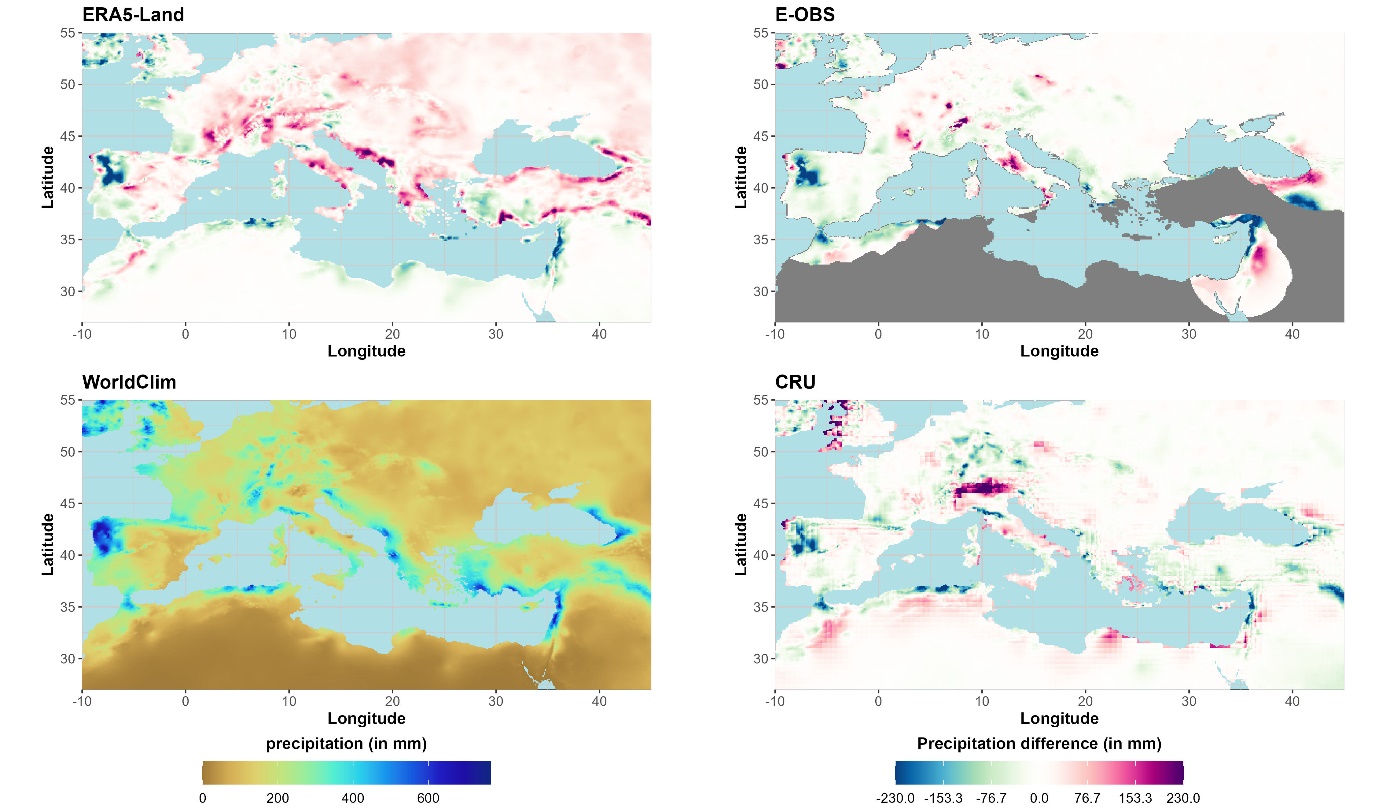
*

*S2.14:* ***BCV-19*** *Precipitation of the coldest quarter. The figure shows the precipitation amounts of the WorldClim dataset (top left) and the precipitation deviations (dataset - WorldClim) of ERA5-Land (bottom left), E-OBS (top right) and CRU (bottom right) for the period 1970-2000 (gray areas: No data).*

*
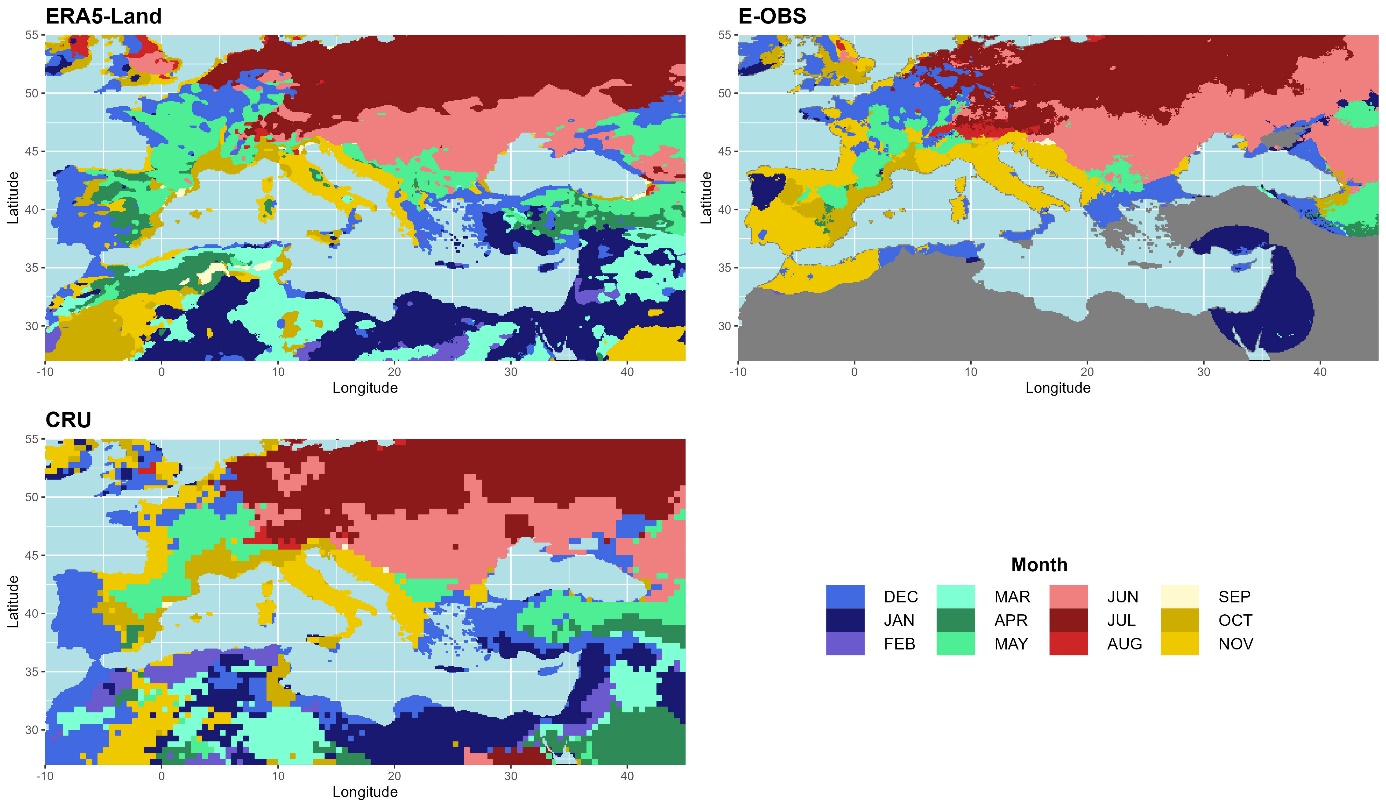
*

*S2.15:* ***BCV-13*** *Precipitation of the wettest month. The wettest month for ERA5-Land (top left), E-OBS (top right) and CRU (bottom left) for the period 1970-2020 (gray areas: No data).*

*
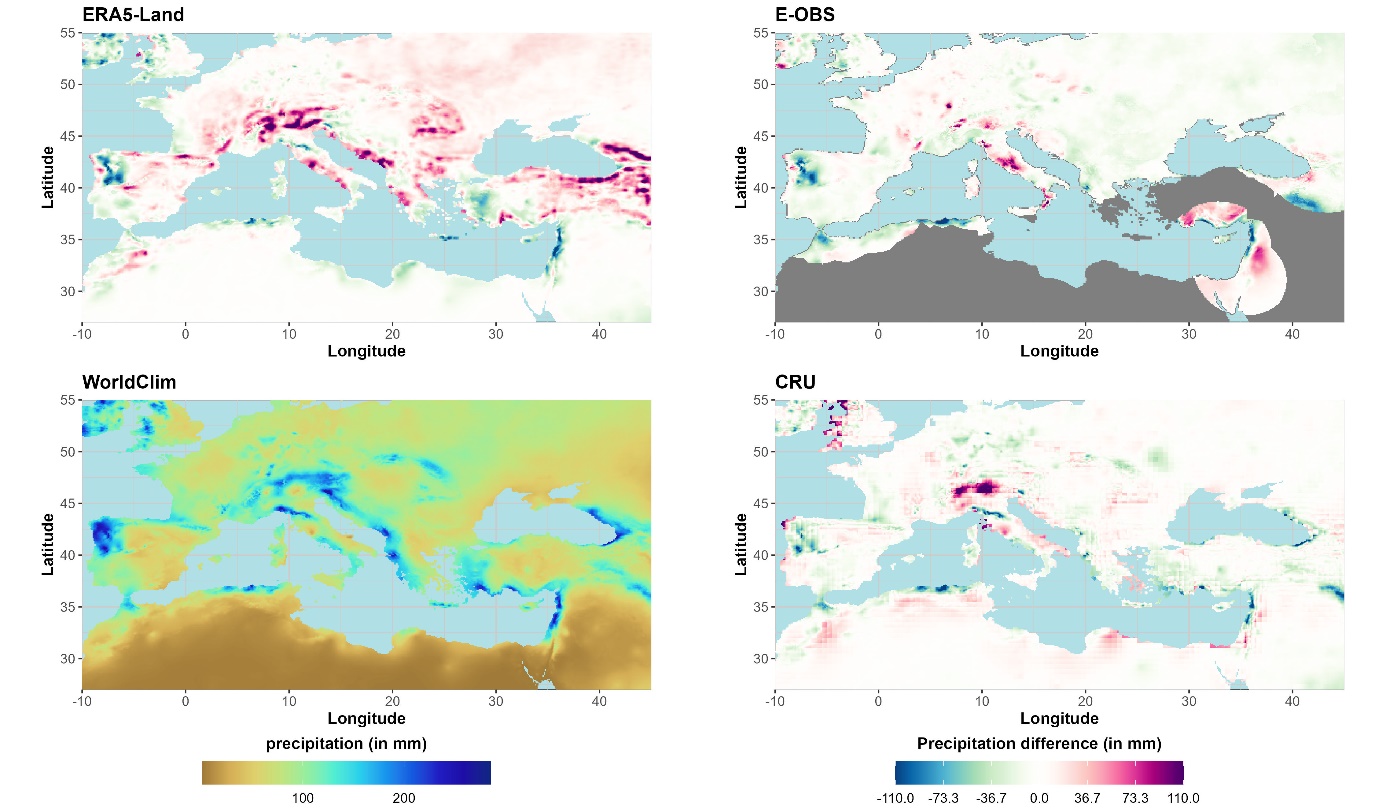
*

*S2.16:* ***BCV-13*** *Precipitation of the wettest month. The figure shows the precipitation amounts of the WorldClim dataset (top left) and the precipitation deviations (dataset - WorldClim) of ERA5-Land (bottom left), E-OBS (top right) and CRU (bottom right) for the period 1970-2000 (gray areas: No data).*

*
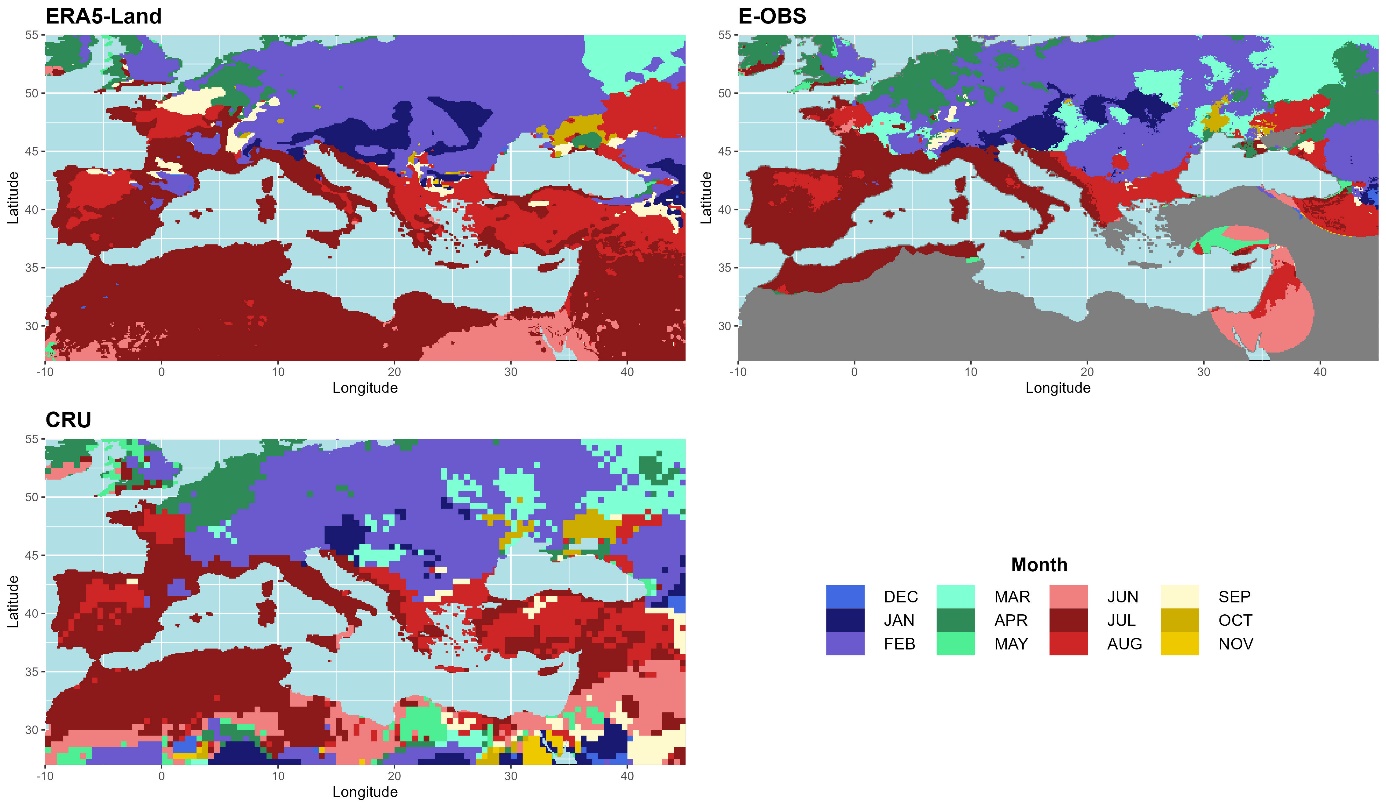
*

*S2.17:* ***BCV-14*** *Precipitation of the driest month. The driest month for ERA5-Land (top left), E-OBS (top right) and CRU (bottom left) for the period 1970-2020 (gray areas: No data).*

*
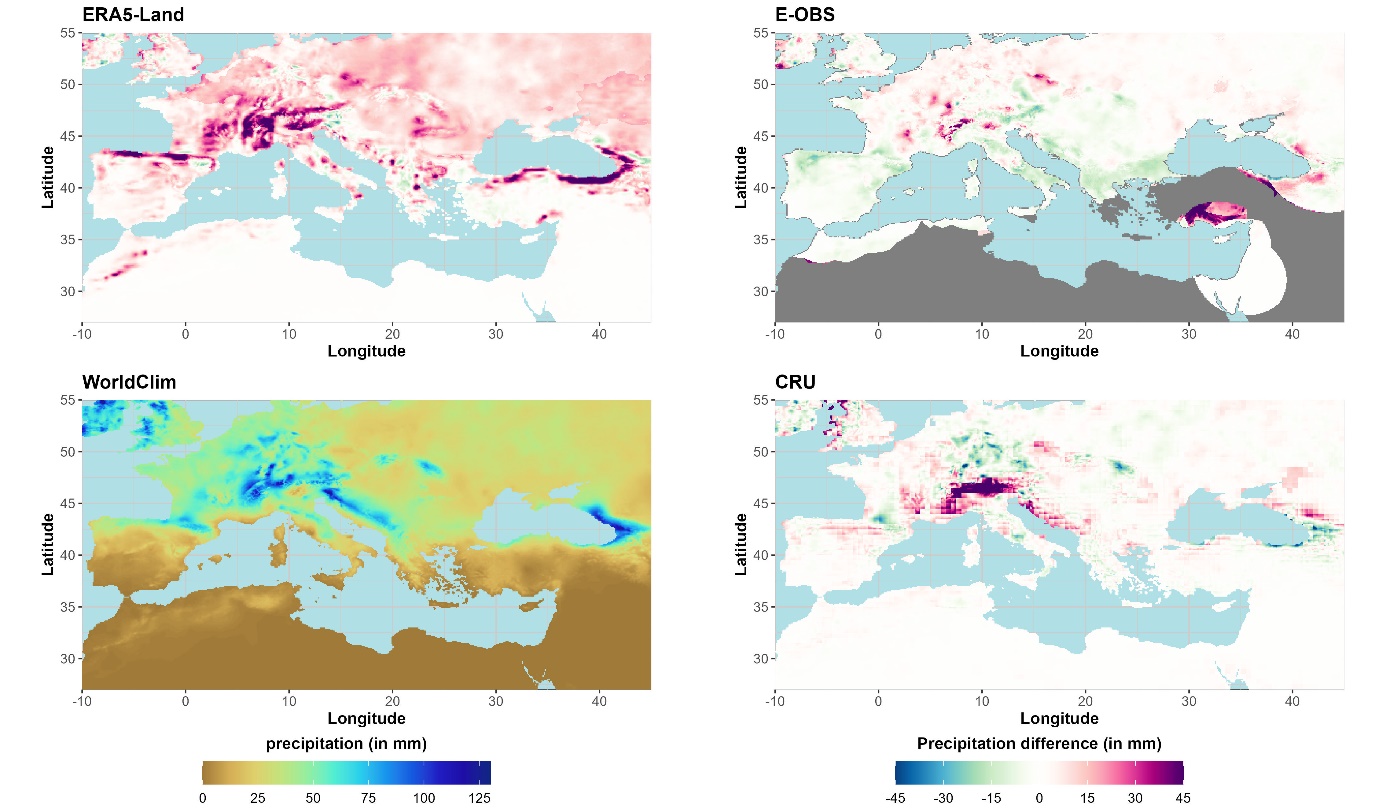
*

*S2.18:* ***BCV-14*** *Precipitation of the driest month. The figure shows the precipitation amounts of the WorldClim dataset (top left) and the precipitation deviations (dataset - WorldClim) of ERA5-Land (bottom left), E-OBS (top right) and CRU (bottom right) for the period 1970-2000 (gray areas: No data).*
